# Infections and cognitive function, depression, and frailty: a cross-sectional study in the longitudinal aging study in India (LASI)

**DOI:** 10.1186/s12889-025-23490-w

**Published:** 2025-07-02

**Authors:** Georgia R. Gore-Langton, Kathryn E. Mansfield, Prithvee Ravi, Arrisonia Doubatty, Suvarna Alladi, Sanjay Kinra, Charlotte Warren-Gash

**Affiliations:** 1 Department of Non-Communicable Disease Epidemiology, London School of Hygiene and Tropical Medicine, London, UK; 2 School of Health and Care Sciences, University of Lincoln, Lincoln, UK; 3 National Institute of Mental Health and Neurosciences, Bengaluru, India

**Keywords:** Infections, Cognitive function, Depression, Frailty, India, Cross-sectional survey

## Abstract

**Introduction:**

Infections may be associated with an increased risk of poor brain health and frailty among older people, but evidence from low-and-middle-income countries (LMICs) is limited. We aimed to investigate associations between nine infections and cognition, depression, and frailty in India.

**Methods:**

We conducted a cross-sectional study using data from Wave 1 (2017–2019) of the Longitudinal Aging Study in India (LASI) survey of adults (≥ 45years) from 35 of India’s 36 states and union territories. Data were collected via face-to-face interviews and direct health measurements. We investigated the association between nine infections, either self-reported ever (periodontal disease) or in the two years before interview (jaundice/hepatitis, malaria, tuberculosis, typhoid, chikungunya, diarrhoea/gastroenteritis, dengue, urinary tract infection [UTI]), and measured global cognitive function, depression, and frailty. We used survey-weighted multivariable logistic regression to compare odds of impaired cognition, depression, and frailty in people with and without infections, overall and for individual infections.

**Results:**

We included 64,682 respondents; median age 59 years (IQR:50–67), 53.5% female, 35% reported at least one infection. After controlling for demographic, social/environmental and lifestyle factors, and chronic health conditions, we saw evidence of associations between infection and both depression (OR: 1.28 [95%CI: 1.22–1.35]) and frailty (OR: 1.74 [95%CI: 1.65–1.84]). UTIs were associated with the highest odds of both depression (OR: 1.33 [95%CI: 1.14, 1.55) and frailty (OR: 2.94 [95%CI: 2.51, 3.44]). Reporting at least one infection was associated with reduced odds of impaired cognition (OR: 0.80 [95%CI: 0.74–0.86]).

**Conclusions:**

Our results suggest infections are associated with increased depression and frailty in adults over 45 in India. Our finding of association between reported infections and better cognition, is potentially explained by preferential infection recall in those with better cognition. Longitudinal studies are needed to investigate potential causal links between infections and adverse brain health and frailty and guide interventions to improve the health of older people in India and other LMICs.

**Supplementary Information:**

The online version contains supplementary material available at 10.1186/s12889-025-23490-w.

## Introduction

Population ageing is occurring rapidly in low- and middle-income countries (LMICs); it is estimated that by 2050, 22% of the world’s population will be aged over 60 years (2.1 billion people) [1]. Conditions affecting brain health and function are common in older age, with dementia and depression affecting 5% and 7% of individuals aged over 60 worldwide [2]. Cognitive impairment refers to problems with memory or thinking; is it demonstrated by a low score on a standardised cognitive assessment. Cognitive impairment is not dementia in itself but can be a sign of the disease which will eventually cause dementia. Frailty, or an individual’s overall resilience and capacity to recover after health problems, is also strongly linked to age. Poor brain health and frailty are detrimental for individuals, their community and wider society, and have substantial economic costs [3]. Better understanding of risk factors for poor brain health and frailty in older age will help to inform prevention strategies, which are especially important in settings with limited treatment options.

Infections may affect future risk of poor brain health. There is strong evidence that infections directly affecting the central nervous system (CNS), such as viral encephalitis, are associated with impaired cognition (which can be an early sign of dementia) and dementia [4]. While biologically plausible that non-CNS infections are detrimental to brain health, via systemic inflammation [5, 6] the links are less well understood. Non-CNS infections such as pneumonia and urinary tract infections (UTIs) are associated with increased risk of dementia [7], however, any links with an increased risk of cognitive impairment are less clear. Limited evidence suggests a dose-response relationship between the number of infections and subsequent risk of a mood disorder [8, 9]. Increased frailty or functional decline has also been associated with severe non-CNS infections such as community-acquired bacteraemia and repeat infections such as recurrent UTIs [10, 11]. Despite India’s large infection burden and rapidly ageing population, infection-associated risks of poor brain health and frailty have not been systematically investigated in this setting [12]. 

We therefore aimed to conduct a cross-sectional study of the non-CNS infection associated risk of impaired cognition, depression, and frailty among adults ≥ 45 years using survey data from Phase 1 of the Longitudinal Aging Study in India (LASI).

## Methods

### Study population

Our study used data from Wave 1 of LASI (April 2017 to December 2018) and included respondents aged 45 years and older from 35 of India’s 36 states/union territories (Sikkim not included). The LASI sampling frame included only community-dwelling individuals (excluding people in collective institutional living arrangements). LASI adopted a multistage stratified area probability cluster sampling design: three-stage sampling in rural areas, and four-stage sampling in urban areas [13]. Respondents were asked to provide written informed consent in their preferred language. The consent form was read to respondents who were unable to read and they were asked to provide signature or inked fingerprint as signature.

### Data collection methods

Interviews were conducted by trained field investigators and covered topics including demographics, work, retirement and pensions, health, and family and social networks. Proxy interviews were conducted for participants who were incapable of giving a full interview. Only participants who were interviewed directly were included in our study. Trained health investigators took various other measurements including anthropometrics, blood pressure, and dried blood spots.

### Exposures

Our primary infection exposure was reporting at least one of any of nine types of infections: periodontal disease, jaundice/hepatitis, malaria, tuberculosis (TB), typhoid, chikungunya, diarrhoea/gastroenteritis, dengue, and urinary tract infection (UTI). Respondents were asked whether they had ever had periodontal disease, and whether they had had each of the other eight infections during the past two years. We also investigated each individual infection as a separate exposure in secondary analyses.

### Outcomes

We focussed on three outcomes: (i) impaired cognition; (ii) depression; and (iii) frailty. Cognition was measured under three domains as part of the regular cognition module of the Health and Retirement Study (HRS): memory (score 0–20 based on the sum of immediate recall of a 10-word list (score 0–10) and delayed recall of a 10-word list which is read to the respondent and recall is tested after time spent answering other survey questions); orientation (score 0–4 for orientation to time [year, month, date, day]); and executive function (score 0–6 was the sum of the score of ability to subtract numbers and binary [correct/incorrect] score of ability to count backwards from 20) [14, 15]. The final score for each of the three domains was expressed as a Z-score, standardised using the study population mean and standard deviation. The average of the three Z-scores was calculated, giving a Global Cognition Function Score Z-score (GCF Z-score) for each individual, with positive and greater values indicating better cognitive function. The primary cognition outcome was a GCF Z-score in the bottom 10th percentile for an individual’s 10-year age group, classified as the binary outcome of having impaired cognition [14, 16]. GCF Z-score as a continuous outcome was investigated in secondary analyses.

Depression was measured via ten items from the Centre for Epidemiology Studies Depression Scale (CES-D), validated for screening in older adults [17]. Seven items concerned negative symptoms, and three items concerned positive symptoms (Table S1). Each item was given a score of 0–3 to reflect frequency of symptoms during the past week (0: “rarely or never”, 3:; “most or all of the time”), with scoring reversed for positive symptoms, to give a range of total scores between 0 and 30 (with a higher score indicating more depressive symptoms). In the primary analyses, participants were considered to have the binary outcome ‘depression’ if for four or more of the questions they reported scores of 2 or 3 (“often” or “most or all of the time” for the negative symptoms or “sometimes” or “rarely or never” for the positive symptoms). We investigated total CES-D score as a continuous outcome in secondary analyses.

Frailty was measured by calculating a frailty index, which was the mean of 37 binary (yes [1]/no [0]) responses to items covering five domains: activities of daily living (ADLs); co-morbidities; instrumental activities of daily living (IADL); mobility; and self-reported health (Table S2) [18]. Frailty index values ranged from 0 to 0.89, with individuals with a frailty index ≥ 0.25 classified as ‘frail’, and those with scores less than 0.25 as ‘not-frail’ [19, 20]. We considered frailty index as a continuous outcome in a secondary analysis.

### Covariates

We considered the following variables as potential confounders: age, sex, social and environmental co-variates (poverty, caste, marital status, social contact, education level, household cooking fuel, sanitation level), lifestyle co-variates (alcohol, smoking, body mass index [BMI] categorised as per guidelines for South Asians into ‘underweight’ [BMI < 18.5], ‘normal’ [BMI ≥ 18.5 & <23], ‘overweight’ [BMI ≥ 23 & <25], ‘obese’ [BMI ≥ 25]), [21] chronic health conditions (controlled for via the frailty index, which contains information on co-morbidities and activities of daily living), and anaemia [15]. Interaction with the health care system (whether, or not, the respondent had received health care or consulted with a health care provider in the past year), was investigated as an effect modifier. Details for each covariate are provided in Table S3.

### Statistical analysis

#### Main analyses

We initially described the non-survey-weighted descriptive characteristics (n [%] for categorical variables, and median [interquartile range [IQR]] for continuous variables) for individuals with and without at least one infection. We also described individuals who were and were not included in each of the main analyses (infection-cognition, infection-depression, infection-frailty).

We survey weighted data at the individual level to account for unequal selection probabilities of households, and therefore individuals, differential non-response rates, and to bring the sample in line with key socio-demographic variables within the reference population. We used the Stata svy commands to survey weight, and R survey package for all further analyses [22, 23]. We used logistic regression with robust standard errors to estimate odds ratios (OR) and 95% confidence intervals (95% CI) for each categorical outcome (impaired cognition, depression, frailty), and linear regression to estimate the difference in continuous outcomes (GCF Z-score, depression score, frailty index), in people with at least one infection relative to those without an infection. All analyses were complete case analyses.

For each outcome (cognition, depression, frailty), we initially ran minimally adjusted models controlling for sex and age. We then followed with sequential models additionally adjusting for: (1) social and environmental factors; (2) lifestyle factors; and (3) for cognition and depression outcomes only, frailty as a means of capturing chronic health conditions (Table S3). We tailored our modelling approach appropriately for each of the three outcomes. We excluded weekly social contact with friends in the frailty analysis as social contact was not considered to be associated with increased frailty. We included categorical BMI in the depression analysis but not in the frailty analysis as one of the items in the frailty index is “difficulty eating”, which is likely to be related to BMI (Table S2). We also did not include BMI in our analyses of impaired cognition, as low BMI may be a consequence of impaired cognition [24]. We adjusted for frailty in fully-adjusted cognition and depression analyses to additionally adjust for chronic health conditions as the co-morbidities domain of the frailty index captures information on nine health conditions (Table S2). We did not adjust for chronic health conditions in analyses with frailty as an outcome as chronic comorbidities were included in the frailty outcome definition.

#### Sensitivity analyses

To test how robust our findings were, we repeated our main analysis including: (1) depression as a confounder in the association between at least one infection and impaired cognition and frailty; (2) impaired cognition as a confounder in the association between infection and frailty; and (3) anaemia as a confounder in the association between malaria and each of impaired cognition, depression, and frailty.

#### Secondary analyses

In secondary analyses, we investigated each of the nine infections separately, comparing individuals with each of the nine infections to those without that specific infection on the same outcomes investigated in the main analysis (i.e., categorial and continuous measures of cognition, depression and frailty using logistic or linear regression as appropriate).

We also investigated whether the association between infections and each of impaired cognition, depression, and frailty, was: (1) stronger the more infections an individual reported; or (2) modified by interaction with the health system, categorical age (45–59; 60–75; 75+), or sex.

**Table 1:** Unweighted characteristics of the LASI respondents by infection status (results are N (%) unless otherwise stated)

Characteristic	No infection, *N* = 43,135^1^	At least one infection, *N* = 21,547^1^
**Age (yrs)**	58.00 [50.00–67.00]	59.00 [51.00–66.00]
**Sex**		
Male	20,505 (47.5)	9,549 (44.3)
Female	22,630 (52.5)	11,998 (55.7)
(Missing)	0 (0.0)	0 (0.0)
**International Poverty Line** ^**2**^		
Above int poverty line	35,981 (83.4)	>17,724 (>82.3)
Below int poverty line	7,154 (16.6)	3,773 (17.5)
(Missing)	0 (0.0)	<50 (<0.1)
**Caste** ^**3**^		
Scheduled caste (SC)/Scheduled tribe (ST)/Other backwards tribe (OBT)	30,591 (70.9)	15,808 (73.4)
No caste or other	12,157 (28.2)	5,640 (26.2)
(Missing)	387 (0.9)	99 (0.5)
**Marital status**		
Married/partnered	>32,399 (>75.1)	16,342 (75.8)
Not married	10,686 (24.8)	5,205 (24.2)
(Missing)	<50 (<0.1)	0 (0.0)
**Weekly contact with friends/family** ^**4**^		
No	36,505 (84.6)	18,653 (86.6)
Yes	6,110 (14.2)	2,650 (12.3)
(Missing)	520 (1.2)	244 (1.1)
**Education** ^**5**^		
Never attended school	18,814 (43.6)	11,472 (53.2)
Less than primary	4,963 (11.5)	2,426 (11.3)
Primary	5,865 (13.6)	2,677 (12.4)
Middle school	>4,350 (>10.1)	1,888 (8.8)
Secondary	6,331 (14.7)	2,263 (10.5)
Diploma/grad/post-grad/prof qualification	2,860 (6.6)	821 (3.8)
(Missing)	<50 (<0.1)	0 (0.0)
**Household uses clean cooking fuel** ^**6**^		
No	17,889 (41.5)	11,423 (53.0)
Yes	24,395 (56.6)	9,732 (45.2)
(Missing)	851 (2.0)	392 (1.8)
**Household has Improved Sanitation** ^**7**^		
No	10,546 (24.4)	7,076 (32.8)
Yes	31,732 (73.6)	14,068 (65.3)
(Missing)	857 (2.0)	403 (1.9)
**Drinks Alcohol** ^**8**^		
Never	38,424 (89.1)	19,230 (89.2)
Infrequently	2,472 (5.7)	1,417 (6.6)
Frequently	1,959 (4.5)	806 (3.7)
(Missing)	280 (0.6)	94 (0.4)
**Ever smoked**		
No	35,514 (82.3)	17,011 (78.9)
Yes	7,336 (17.0)	4,432 (20.6)
(Missing)	285 (0.7)	104 (0.5)
**BMI Category**		
Underweight (BMI < 18.5)	6,440 (14.9)	4,525 (21.0)
Normal (BMI > = 18.5 & <23)	14,272 (33.1)	7,679 (35.6)
Overweight (BMI > = 23 & <25)	6,166 (14.3)	2,746 (12.7)
Obese (BMI > = 25)	12,192 (28.3)	5,043 (23.4)
(Missing)	4,065 (9.4)	1,554 (7.2)
**Anaemia** ^**9**^		
No	>42,365 (>98.2)	>19,738 (>91.6)
Yes	786 (1.8)	1,759 (8.2)
(Missing)	<50 (<0.1)	<50 (<0.1)
**Medical visit** ^**10**^		
No	21,592 (50.1)	8,307 (38.6)
Yes	21,158 (49.1)	13,082 (60.7)
(Missing)	385 (0.9)	158 (0.7)

^1^Median [25-75%]; n (%)

^2^Whether the household falls above or at/below the international poverty line as defined by the World Bank which is currently set at $1.90 per person per day in 2011 purchasing power parity (PPP) dollars

^3^SC/ST/OBC population is largely deprived of privileges, historically and socially marginalized, and vulnerable

^4^At least weekly contact with any of their friends or relatives (not including those they live with) in person

^5^Less than primary: standard 1–4, Primary: standard 5–7, Middle: standard 8–9, Secondary: standard 10–12

^6^Clean cooking fuel includes housing respondent reporting their main source of cooking fuel is liquefied petroleum gas (LPG), biogas, or electric

^7^Improved sanitation includes flush or pour flush toilet that flushes to a piped sewer system, flushes to a septic tank, or flushes to pit latrine, or if the housing respondent reports a twin pit/composting toilet or pit latrine, and the housing respondent reports that the household does not share their toilet facility with any other households

^8^Infrequently drinks alcohol: drinks less than three days per month. Frequently drinks alcohol: drinks one or more days per week

^9^Whether the respondent has had anaemia in the past two years

^10^Whether the respondent received health care from or consulted with a health care provider (including home visits) in the past year (i.e. outpatient visits)

^11^NB: to prevent disclosure we have used primary suppression to directly suppress cells with small counts (i.e. <50), and secondary suppression to suppress additional cells that do not have small counts themselves but which need to be suppressed to protect the values in the primarily suppressed cells

## Results

We identified a total of 82,650 surveyed individuals, 72,262 participated in individual interviews, 65,575 of whom where aged 45 years and over. Proxy interviews were conducted for 694 participants who were incapable (cognitively or physically) to give a full interview. Excluding these participants resulted in a study population of 64,682 respondents (Figure S1).

Median age was 59 years (IQR:50–67), and 53.5% were women. Accounting for survey weighting, 35.3% (95% CI: 34.8–35.8) of participants reported at least one infection. The unweighted characteristics of those with at least one infection and without infection are shown in Tables 1 and by infection status and outcome in Table S4. A larger proportion of those with an infection than those without an infection had received no, or less than, primary level education (infection 64.5%, no infection 55.1%), were anaemic (infection 8.2%, no infection 1.8%). Lower proportions of those with at least one infection than those without used “clean” cooking fuels (infection 45.2%, no infection 56.6%) and had access to improved sanitation (infection 65.3%, no infection 73.6%). Other characteristics such as age, sex, caste, marital status, and weekly contact with friends/family were similar by infection status. The unweighted demographic characteristics of respondents included in the fully adjusted analyses for each outcome were similar to those who were excluded due to not having complete case data (Tables S5, S6, S7).

The most common infection reported was periodontal disease (15.7%), followed by diarrhoea (13.4%), malaria (8.5%), typhoid (5.8%), jaundice (2.7%), chikungunya (2.6%), UTIs (2.1%), dengue (1.1%), and TB (0.9%). Of those reporting an infection, 23.4% reported just one infection, 8.2% had two infections, and 3.7% of respondents reported three or more infections (Table S8).

### Cognition

**Fig. 1:**
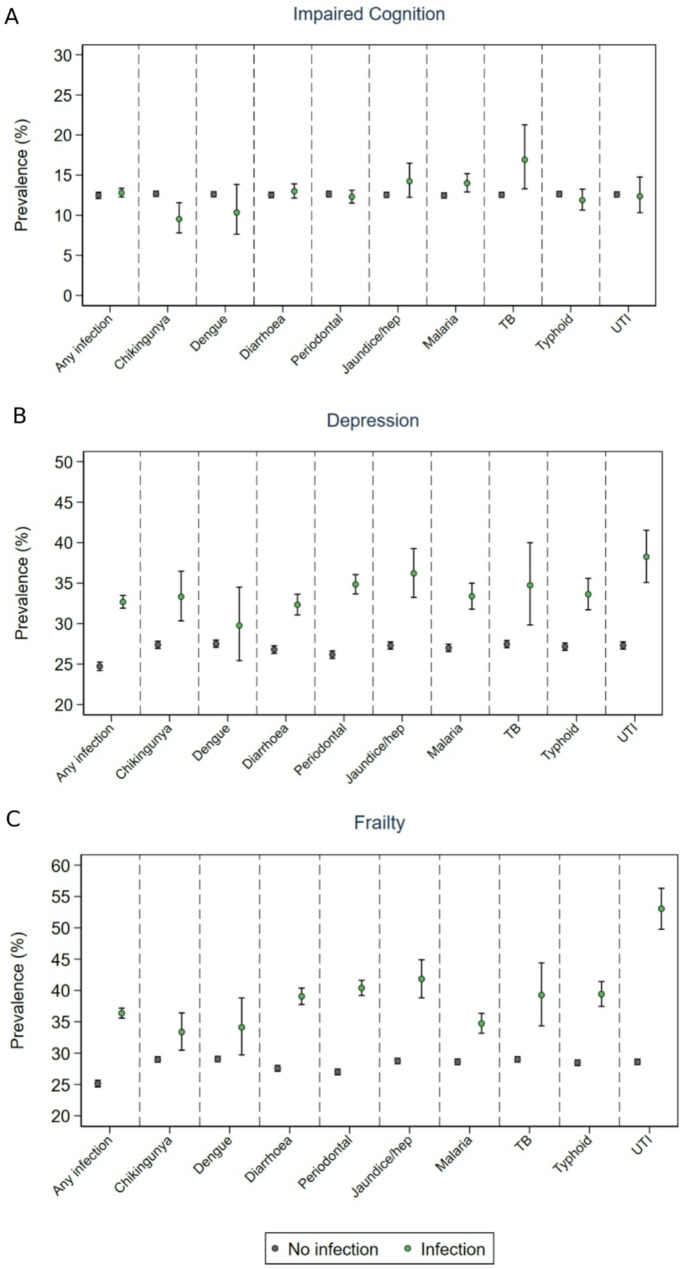
Prevalence of (**A**) impaired cognition, (**B**) depression, (**C**) frailty by infection status (any infection and each individual infection)

**Fig. 2:**
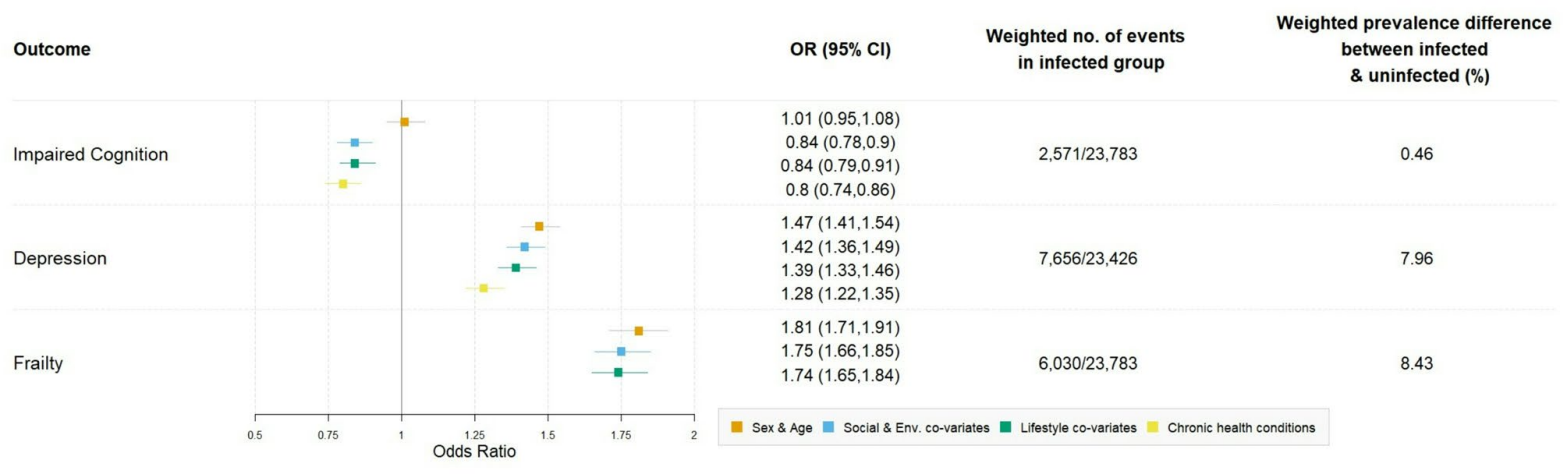
Association between having at least one infection and impaired cognition, depression, and frailty. Social and environmental co-variates include; poverty, caste, marital status, social contact, education level, household cooking fuel, sanitation level; lifestyle co-variates include alcohol, smoking, BMI, and chronic health conditions adjusts for frailty, which contains information on co-morbidities as well as activities of daily living, hence this model is not run for the outcome of frailty

The overall weighted prevalence of impaired cognition (bottom 10% of GCF Z-scores in 10-year age group) was 10.5% (95% CI: 10.2, 10.8), similar among those with at least one infection 10.8% (95% CI: 10.3, 11.3) and among those without an infection 10.3% (95% CI:10.0, 10.7) (Fig. 1). In the fully adjusted model (adjusted for age, sex, social and environmental factors, lifestyle factors, frailty), having at least one infection was associated with reduced odds of impaired cognition, OR: 0.80 (95%CI:0.74–0.86), (Fig. 2, Table S9). Mean GCF Z-score was − 0.06 (95%CI: -0.05, 0.08) among those with at least one infection and 0.01 (95% CI: 0.01, 0.02) among those without an infection, indicating a higher cognition among those without infection. However, the fully adjusted analyses revealed the reverse, having an infection was associated with a 0.04 (95% CI: 0.03, 0.05) higher mean GCF Z-score relative to the uninfected, indicating a higher level of cognitive function among those reporting an infection (Table S10).

Impaired cognition was lower among those reporting malaria, diarrhoea, typhoid, and periodontal disease, than among those without each infection (Table S11). In contrast, impaired cognition was higher among those reporting TB (OR: 1.50 [95% CI: 1.07, 2.10]) (Table S11), corresponding to a mean reduction in GCF Z-score of 0.11 (95% CI: -0.17, -0.04) (Table S12). We saw no evidence of association between jaundice, UTIs, chikungunya or dengue and impaired cognition (Table S11).

### Depression

Overall, 27.5% (95% CI: 27.1, 28.0) of participants were classified as depressed, the proportion was higher among those with at least one infection, 32.7% (95% CI: 31.9, 33.5), than among those without an infection, 24.7% (95% CI: 24.2, 25.3) (Fig. 1). Those reporting at least one infection were more likely to be classified as depressed (OR 1.28, 95% CI: 1.22–1.35), than those without an infection in fully adjusted models (Fig. 2, Table S13). Among those with at least one infection, mean depression score was 10.39 (95% CI: 10.32, 10.46), indicating more depressive symptoms than the uninfected with a mean score of 9.33 (95% CI: 9.28, 9.38). Similarly, after fully adjusting for age, sex, social and environmental factors, lifestyle factors, and frailty, the mean depression score was 0.70 (95% CI: 0.62, 0.79) units higher among those with at least one infection than those uninfected (Table S14).

Risk of depression was higher with each individual infection, except TB and dengue (Table S11). At particular risk were those reporting chikungunya (OR: 1.31 [95% CI: 1.12, 1.53]), UTI (OR: 1.33 [95% CI: 1.14, 1.55]), periodontal disease (OR: 1.30 [95% CI: 1.22, 1.38], and jaundice (OR: 1.28 [95% CI: 1.11, 1.48]). This elevated risk corresponded to a mean increase in depression score ranging from 0.28 (95% CI: 0.16, 0.39) among those with diarrhoea, to 1.02 (95% CI: 0.76, 1.28) among those with chikungunya (Table S12).

### Frailty

In total, 19.9% (95% CI: 19.5, 20.3) of participants were frail, with a higher proportion among those with at least one infection (25.4%, 95% CI: 24.7, 26.1), than among those without an infection (16.9%, 95% CI: 16.5, 17.4) (Fig. 1). Those who had at least one infection were more likely to be frail than those without an infection, (fully adjusted OR: 1.74, [95% CI: 1.65, 1.84]) (Fig. 2, Table S15). Median frailty index was 0.135 (IQR: 0.054, 0.270) among those with infection and 0.081 (IQR: 0.027, 0.189) among those without. In the fully adjusted model (adjusted for age, sex, social and environmental factors, lifestyle factors), mean frailty index was 0.039 (95% CI: 0.036, 0.042) higher among those infected than those uninfected, indicating higher frailty, Table S16.

We saw strong evidence of increased frailty associated with each of the nine infections. At particular risk were UTIs (OR: 2.94 [95% CI: 2.51, 3.44]), jaundice (OR: 2.01 [95% CI: 1.72, 2.36), periodontal disease (OR: 1.92 [95% CI: 1.79, 2.06]) (Table S11). The greatest mean increase in frailty index, 0.082 (95% CI: 0.072, 0.092), was associated with having a UTI (Table S12).

### Sensitivity analyses

There was no evidence that depression confounded the association between infection status and either cognition or frailty. Nor was there evidence that cognition confounded the association between infections and frailty (Table S17). Additionally, adjusting for anaemia in the analysis investigating the association between malaria and frailty attenuated the odds ratio from 1.28 (95% CI: 1.17, 1.40) to 1.17 (95% CI: 1.07–1.29), but the 95% CIs for the two estimates overlapped. Controlling for anaemia did not change the association between malaria and impaired cognition or frailty (Table S17).

### Secondary analyses

#### Number of infections

Frailty increased with a respondent’s total number of reported infections, compared to those without an infection: OR: 1.69 (95% CI: 1.60-1.791) among those with 1–3 infections, and OR: 3.60 (95% CI: 2.85, 4.53) among those reporting 4 or more infections (Table S18). There was no evidence of a difference in risk of impaired cognition or depression associated with a respondent’s total number of reported infections.

#### Effect modification

There were two examples of evidence of modification of the association between at least one infection and frailty: (1) the association was greater among those who had not attended a medical visit in the past year (OR: 1.98 [95% CI: 1.81, 2.17]), than among those who had (OR: 1.53 [95% CI: 1.43, 1.64], *p*-value ^interaction^: <0.001 Table S19); and (2) younger respondents were at higher risk of infection associated frailty (45–59 yrs OR: 1.91 [95% CI: 1.74, 2.09], 60–75 years OR: 1.66 [95% CI: 1.54, 1.79], 75 + year OR: 1.38 [95% CI: 1.20, 1.58], *p*-value ^interaction^: <0.001, Table S20). There was no evidence of effect modification of the association between infections and either impaired cognition or depression by medical visit attendance (Table S19), age (Table S20), or sex (Table S21), nor of effect modification of the association between infections and frailty by sex.

## Discussion

### Statement of principal findings

In a nationwide representative sample of those 45 years and older in India, we found self-reporting at least one of nine infections to be associated with increased frailty and depression. We found evidence of this association across a range of infection types. Only the chronic infection TB was associated with increased risk of impaired cognition. UTIs, which are typically acute, were strongly associated with both frailty and depression. The total number of infections a respondent reported was strongly associated with increased frailty, but not impaired cognition or depression. The risk of impaired cognition was lower among those reporting an infection than among those not reporting an infection, implying a protective effect of infections against impaired cognition. However, this counter-intuitive result may be explained by under-reporting of recent infections in people with impaired cognition. Respondents with TB, the only infection associated with impaired cognition, may have more accurate recall considering it is a longer-term infection requiring extensive drug regimens, than recall of shorter-term more acute infections such as UTIs, which have previously been found to be associated with increased dementia [7]. 

### Strengths

Due to a large sample size and weighting, the LASI Wave 1 dataset is nationally representative of India’s population aged 45 years and older. Using this dataset allowed us to investigate relationships between infections and brain health outcomes in an under-represented population. Most previous studies investigating similar relationships have been set in high-income countries [7, 9–11] [25]. Our results are strengthened by our outcome measures not being reliant on health seeking behaviour; cognition, depression, and frailty were assessed systematically using validated tools.

### Limitations

However, our study also has limitations. Unlike the verified outcome measures, the data collected on our exposures of interest (infections) relied on self-reporting by respondents and are therefore vulnerable to misclassification. LASI contains no laboratory confirmed diagnostic data nor information on duration or severity of infections. The absence of longer-term history of infection data (longer than two years for all infections except periodontal disease) may have resulted in the misclassification of individuals with longer-term previous infections as unexposed, therefore under estimating associations between infections and poor brain health. In our analyses we considered hepatitis and diarrhoea as having an infectious cause; while this is likely in the setting, there are possible non-infectious causes of both [26, 27]. Excluding participants who were not cognitively or physically able to give full interviews (*n* = 694) may have excluded a sub-group at particular risk of infections and poor brain health.

Our analyses were conducted in accordance with Harmonized LASI guidance, using individual post-stratification weights provided to account for unequal selection probabilities and differential non-response. Although robust standard errors were used, the lack of available primary sampling unit and strata variables in LASI mean that the effects of clustering may not be fully accounted for.

While we made efforts to account for the wide range of confounders ascertained in the LASI questionnaire, it is possible that residual confounding remained through unmeasured confounders such as potential genetic predispositions to infections and poor brain health. Adjusting for frailty, which included both measures of co-morbidities and activities of daily living, in the depression and impaired cognition models may have resulted in overadjustment. If the frailty index included mediators, this could lead to bias towards the null. Overadjustment could also have resulted in collider bias if the frailty index was driven by other unmeasured risk factors for the outcome [28]. Nevertheless, given the consistency of associations between the models adjusting for social, environmental and lifestyle co-variates, and the models additionally including adjustment for frailty, this is unlikely to have majorly affected results. In secondary analyses investigating each of the nine infections as the exposure for each of the three outcomes, involved a large number of statistical models and therefore increases the risk of a false-positive finding. In addition, the cross-sectional nature of the LASI Wave 1 data precluded the temporal analyses required to rule out reverse causation and strengthen any conclusions regarding causal associations and pathways between infections and brain health.

### Findings in the context of existing literature

Most of the nine infections investigated in our analyses were infections that typically present acutely. Whilst data from India and LMICs on the relationship between acute infections and brain health outcomes are scarce, there is a body of evidence from high-income countries [7, 9–11] [25]. Contrary to our reported reduction in risk of impaired cognition among those reporting an infection, results from the UK have shown an increase in dementia risk after an acute infection [7]. Evidence of association was particularly strong for severe infections leading to hospital admission. Longitudinal analyses of cohort data from Finland and the UK also reported an increase in risk of incident dementia associated with hospitalisation for any of the 925 infections investigated, and concluded their findings suggested systemic inflammation rather than specific infection or pathogen was driving dementia risk [25]. 

Few studies have investigated the association between non-CNS infections and mental health outcomes, including depression. Consistent with our findings, two large Danish population studies reported an increase in the risk of diagnosis with a mood disorder associated with both infections treated in out-patient settings [9], typically milder, and infections resulting in hospitalisation [8]. Similarly, studies investigating chronic infections (including HIV, leishmaniasis and multidrug-resistant-TB) and mental health/ depression, have also found an association [29–31]. 

Evidence of associations between infections and incident or worsening frailty is limited and often conflicting. Severe non-CNS infections such as community-acquired bacteraemia have been associated with increased frailty or functional decline [10], as have repeat infections such as recurrent UTI [11]. However, there is limited systematic investigation of frailty as an outcome across a range of infections, nor in LMIC settings. There is no LASI validated frailty index level for categorising frailty. We have used a cut off value of ≥ 0.25 to align with the English Longitudinal Study of Ageing, this differs from ≥ 0.21 validated in the Mexican Health and Aging Study [19, 32]. Longitudinal studies recruiting both exposed and comparator groups, and using validated and internationally comparable frailty definitions, are needed to assess whether associations between infections and frailty are causal.

### Explanations and implications for clinicians, policymakers and future research

Studies with clear temporal relationships between infections and brain health outcomes would offer greater confidence in causal associations. However, our study provides rare detail on the association between nine non-CNS infections and three verified brain health outcomes using a large and nationally representative dataset from India, where evidence of such associations is limited.

We saw an association between self-reported infections and increased depression and frailty. Even if this relationship is not causal, it has public health implications. Care providers could offer mental health support or frailty prevention interventions to people upon diagnosis of an infection. Future studies, designed to investigate the temporal relationships between infections and depression/frailty, would help us to understand whether preventing and treating infections could directly reduce the burden of poor brain health. The unexpected protective association between most infections and impaired cognition requires further research with high quality exposure (infection diagnosis/ laboratory-confirmed) data.

The dose-response association between reported number of infections and frailty in our study may provide evidence that general inflammation, rather than a specific microbe, is the driving factor between infections and frailty, as suggested for the relationship between infections and dementia risk [25]. The reported effect modification of the association between infections and frailty by age, with stronger evidence of an association among younger people, is plausibly explained by age-associated frailty risk being so great among older people that it overwhelms other risk factors such as infections. Similarly, risk factors for dementia which have good predictive power among younger people do not perform well among older people [33]. 

## Conclusions

While a cross-sectional association was seen between infections and increased depression and frailty, longitudinal studies are needed to investigate a causal link between infections and adverse brain health, and guide interventions to reduce the burden of impaired cognition, depression, and frailty in India and LMICs more widely.

## Electronic supplementary material

Below is the link to the electronic supplementary material.

Supplementary Material 1

## Data Availability

LASI Wave 1 2017–18 data and related documentation, including fact sheets, national report and executive summary, are publicly available and can be accessed from the website of the Gateway to Global Aging Data (g2aging.org).

## References

[1] World Health Organization. Ageing and health 2021 [Available from: https://www.who.int/news-room/fact-sheets/detail/ageing-and-health accessed 16th May 2024.

[2] World Health Organization. Mental health of older adults 2017 [accessed 1st November 2022.

[3] Wimo A, Seeher K, Cataldi R, et al. The worldwide costs of dementia in 2019. Alzheimer’s Dement. 2023;19(7):2865–73. 10.1002/alz.12901. 36617519 10.1002/alz.12901 PMC10842637

[4] Granerod J, Davies NWS, Ramanuj PP, et al. Increased rates of sequelae post-encephalitis in individuals attending primary care practices in the united kingdom: a population-based retrospective cohort study. J Neurol. 2017;264(2):407–15. 10.1007/s00415-016-8316-8. 27766471 10.1007/s00415-016-8316-8

[5] Darweesh SKL, Wolters FJ, Ikram MA, et al. Inflammatory markers and the risk of dementia and alzheimer’s disease: A meta-analysis. Alzheimers Dement. 2018;14(11):1450–59. 10.1016/j.jalz.2018.02.014. [published Online First: 20180329]. 29605221 10.1016/j.jalz.2018.02.014

[6] Holmes C, Cunningham C, Zotova E, et al. Systemic inflammation and disease progression in alzheimer disease. Neurology. 2009;73(10):768–74. 10.1212/WNL.0b013e3181b6bb95. 19738171 10.1212/WNL.0b013e3181b6bb95 PMC2848584

[7] Muzambi R, Bhaskaran K, Smeeth L, et al. Assessment of common infections and incident dementia using UK primary and secondary care data: a historical cohort study. Lancet Healthy Longev. 2021;2(7):e426–35. 10.1016/s2666-7568(21)00118-5. 34240064 10.1016/S2666-7568(21)00118-5 PMC8245326

[8] Benros ME, Waltoft BL, Nordentoft M, et al. Autoimmune diseases and severe infections as risk factors for mood disorders: A nationwide study. JAMA Psychiatry. 2013;70(8):812–20. 10.1001/jamapsychiatry.2013.1111. 23760347 10.1001/jamapsychiatry.2013.1111

[9] Köhler O, Petersen L, Mors O, et al. Infections and exposure to anti-infective agents and the risk of severe mental disorders: a nationwide study. Acta Psychiatrica Scandinavica. 2017;135(2):97–105. 10.1111/acps.12671. 27870529 10.1111/acps.12671

[10] Dalager-Pedersen M, Thomsen RW, Schønheyder HC, et al. Functional status and quality of life after community-acquired bacteraemia: a matched cohort study. Clin Microbiol Infect. 2016;22(1):78.e1-78.e8. 10.1016/j.cmi.2015.09.006. 10.1016/j.cmi.2015.09.006 26384680

[11] Tang M, Quanstrom K, Jin C, et al. Recurrent urinary tract infections are associated with frailty in older adults. Urology. 2019;123:24–7. 10.1016/j.urology.2018.09.025. [published Online First: 20181006]. 30296501 10.1016/j.urology.2018.09.025 PMC8528015

[12] United Nations Population Division. World population prospects 2019: data booklet (ST/ESA/SER.A/424). New York, NY: United Nations; 2019.

[13] Perianayagam A, Bloom D, Lee J, et al. Cohort profile: the longitudinal ageing study in India (LASI). Int J Epidemiol. 2022;51(4):e167–76. 10.1093/ije/dyab266. 35021187 10.1093/ije/dyab266 PMC9365624

[14] International Institute for Population Sciences (IIPS), NPfHCoEN MHFW, Harvard TH. Chan school of public health (HSPH) and the university of Southern California (USC),. Longitudinal ageing study in India (LASI) wave 1, 2017-18, India report. Mumbai: International Institute for Population Sciences; 2020.

[15] Chien SC, Drystan Y, Jenny P, Yuxuan W, Alden W, Meijer GE, Marco A, Jinkook L, Harmonized LASI, Version A. 3. 2023. 10.25549/h-lasi

[16] Pandav R, Fillenbaum G, Ratcliff G, et al. Sensitivity and specificity of cognitive and functional screening instruments for dementia: the Indo-U.S. Dementia epidemiology study. J Am Geriatr Soc. 2002;50(3):554–61. 10.1046/j.1532-5415.2002.50126.x. 11943056 10.1046/j.1532-5415.2002.50126.x

[17] Andresen EM, Malmgren JA, Carter WB, et al. Screening for depression in well older adults: evaluation of a short form of the CES-D. Am J Prev Med. 1994;10(2):77–84. 10.1016/S0749-3797(18)30622-6. 8037935

[18] Jenkins ND, Hoogendijk EO, Armstrong JJ, et al. Trajectories of frailty with aging: coordinated analysis of five longitudinal studies. Innov Aging. 2022;6(2). 10.1093/geroni/igab059. 10.1093/geroni/igab059 PMC8882228 35233470

[19] Rogers NT, Steptoe A, Cadar D. Frailty is an independent predictor of incident dementia: evidence from the english longitudinal study of ageing. Sci Rep. 2017;7(1):15746. 10.1038/s41598-017-16104-y. [published Online First: 20171116]. 29146957 10.1038/s41598-017-16104-y PMC5691042

[20] Song X, Mitnitski A, Rockwood K. Prevalence and 10-Year outcomes of frailty in older adults in relation to deficit accumulation. J Am Geriatr Soc. 2010;58(4):681–87. 10.1111/j.1532-5415.2010.02764.x. 20345864 10.1111/j.1532-5415.2010.02764.x

[21] Misra A. Ethnic-Specific criteria for classification of body mass index: A perspective for Asian Indians and American diabetes association position statement. Diabetes Technol Ther. 2015;17(9):667–71. 10.1089/dia.2015.0007. 25902357 10.1089/dia.2015.0007 PMC4555479

[22] Survey. analysis of complex survey samples. [program]: R package version 4.4., 2024.

[23] StataCorp. Stata statistical software: release 18. College station. TX: StataCorp LLC; 2023. [program].

[24] Dye L, Boyle NB, Champ C, et al. The relationship between obesity and cognitive health and decline. Proc Nutr Soc. 2017;76(4):443–54. 10.1017/s0029665117002014. [published Online First: 20170911]. 28889822 10.1017/S0029665117002014

[25] Sipilä PN, Heikkilä N, Lindbohm JV, et al. Hospital-treated infectious diseases and the risk of dementia: a large, multicohort, observational study with a replication cohort. Lancet Infect Dis. 2021;21(11):1557–67. 10.1016/s1473-3099(21)00144-4. 34166620 10.1016/S1473-3099(21)00144-4 PMC8592915

[26] Anand AC, Praharaj D. Acute hepatitis in tropics: A rainbow of causes. Indian J Gastroenterol. 2023;42(3):308–10. 10.1007/s12664-023-01403-2. 37300795 10.1007/s12664-023-01403-2

[27] Behera DK, Mishra S. The burden of diarrhea, etiologies, and risk factors in India from 1990 to 2019: evidence from the global burden of disease study. BMC Public Health. 2022;22(1):92. 10.1186/s12889-022-12515-3. 35027031 10.1186/s12889-022-12515-3 PMC8759196

[28] van Zwieten A, Tennant PWG, Kelly-Irving M, et al. Avoiding overadjustment bias in social epidemiology through appropriate covariate selection: a primer. J Clin Epidemiol. 2022;149:127–36. 10.1016/j.jclinepi.2022.05.021. 35662623 10.1016/j.jclinepi.2022.05.021

[29] Alene KA, Clements ACA, McBryde ES, et al. Mental health disorders, social stressors, and health-related quality of life in patients with multidrug-resistant tuberculosis: A systematic review and meta-analysis. J Infect. 2018;77(5):357–67. 10.1016/j.jinf.2018.07.007. 30036607 10.1016/j.jinf.2018.07.007

[30] Pires M, Wright B, Kaye PM, et al. The impact of leishmaniasis on mental health and psychosocial well-being: A systematic review. PLoS ONE. 2019;14(10):e0223313. 10.1371/journal.pone.0223313. 31622369 10.1371/journal.pone.0223313 PMC6797112

[31] Vollmond CV, Tetens MM, Paulsen FW, et al. Risk of depression in people with human immunodeficiency virus: A nationwide Population-based matched cohort study. Clin Infect Diseases: Official Publication Infect Dis Soc Am. 2023;77(11):1569–77. 10.1093/cid/ciad415. 10.1093/cid/ciad415 37467149

[32] García-González JJ, García-Peña C, Franco-Marina F, et al. A frailty index to predict the mortality risk in a population of senior Mexican adults. BMC Geriatr. 2009;9(1):47. 10.1186/1471-2318-9-47. 19887005 10.1186/1471-2318-9-47 PMC2776593

[33] Walters K, Hardoon S, Petersen I, et al. Predicting dementia risk in primary care: development and validation of the dementia risk score using routinely collected data. BMC Med. 2016;14(1). 10.1186/s12916-016-0549-y. 10.1186/s12916-016-0549-y PMC4722622 26797096

