# Supplementary material for: Infections and cognitive function, depression, and frailty: a cross-sectional study in the longitudinal aging study in India (LASI)
Source: BMC Public Health. 2025 Jul 2;25:2244. doi: 10.1186/s12889-025-23490-w (PMC12220408; doi:10.1186/s12889-025-23490-w)
Supplement: Supplementary file 1 — Supplementary Material 1 [file 12889_2025_23490_MOESM1_ESM.docx]

**SUPPLEMENTARY INFORMATION**

**Infections and cognitive function, depression, and frailty: a cross-sectional study in the Longitudinal Aging Study in India (LASI)**

**Figures:**

[Figure 1: Flow chart for inclusion and exclusion into study population for analyses on infections and cognition, depression, and frailty risk 3](#_Toc169526779)

Tables:

[Table 1: CES-D-10 Depression questionnaire 4](#_Toc180164908)

[Table 2: Domains and deficits coded within the frailty index 5](#_Toc180164909)

[Table 3: Structure of the models developed for cognition, depression, and frailty analyses 6](#_Toc180164910)

[Table 4: Unweighted characteristics of the LASI respondents by outcome and infection status (results are N (%) unless otherwise stated) 9](#_Toc180164911)

[Table 5: Unweighted characteristics of the LASI respondents included in and excluded from the cognition analyses due to missing covariate data, by infection status 12](#_Toc180164912)

[Table 6: Unweighted characteristics of the LASI respondents included in and excluded from the depression analyses due to missing covariate data, by infection status 13](#_Toc180164913)

[Table 7: Unweighted characteristics of the LASI respondents included in and excluded from the frailty analyses due to missing covariate data, by infection status 14](#_Toc180164914)

[Table 8: Total number of infections reported by participants 15](#_Toc180164915)

[Table 9: Association between infections and impaired cognition in people with at least one infection and those without an infection 16](#_Toc180164916)

[Table 10: Association between infections and Global Cognitive Function Z-Score in people with at least one infection and those without an infection 17](#_Toc180164917)

[Table 11: Association between each infection and risk of cognitive impairment, depression, frailty 18](#_Toc180164918)

[Table 12: Association between each infection and change in GCF Z-Score, Depression score, Frailty index 19](#_Toc180164919)

[Table 13: Association between infections and depression in people with at least one infection and those without an infection 20](#_Toc180164920)

[Table 14: Association between infections and Depression Score in people with at least one infection and those without an infection 21](#_Toc180164921)

[Table 15: Association between infections and frailty in people with at least one infection and those without an infection 22](#_Toc180164922)

[Table 16: Association between infections and Frailty Index in people with at least one infection and those without an infection 23](#_Toc180164923)

[Table 17: Results of sensitivity analyses 24](#_Toc180164924)

[Table 18: Association between number of infections and impaired cognition, depression, and frailty 25](#_Toc180164925)

[Table 19: Association between attendance at a medical visit during the past year and impaired cognition, depression, and frailty between those with at least one infection relative to those without an infection 26](#_Toc180164926)

[Table 20: Association between categorical age and impaired cognition, depression, and frailty between those with at least one infection relative to those without an infection 26](#_Toc180164927)

[Table 21: Association between sex and impaired cognition, depression, and frailty between those with at least one infection relative to those without an infection 26](#_Toc180164928)


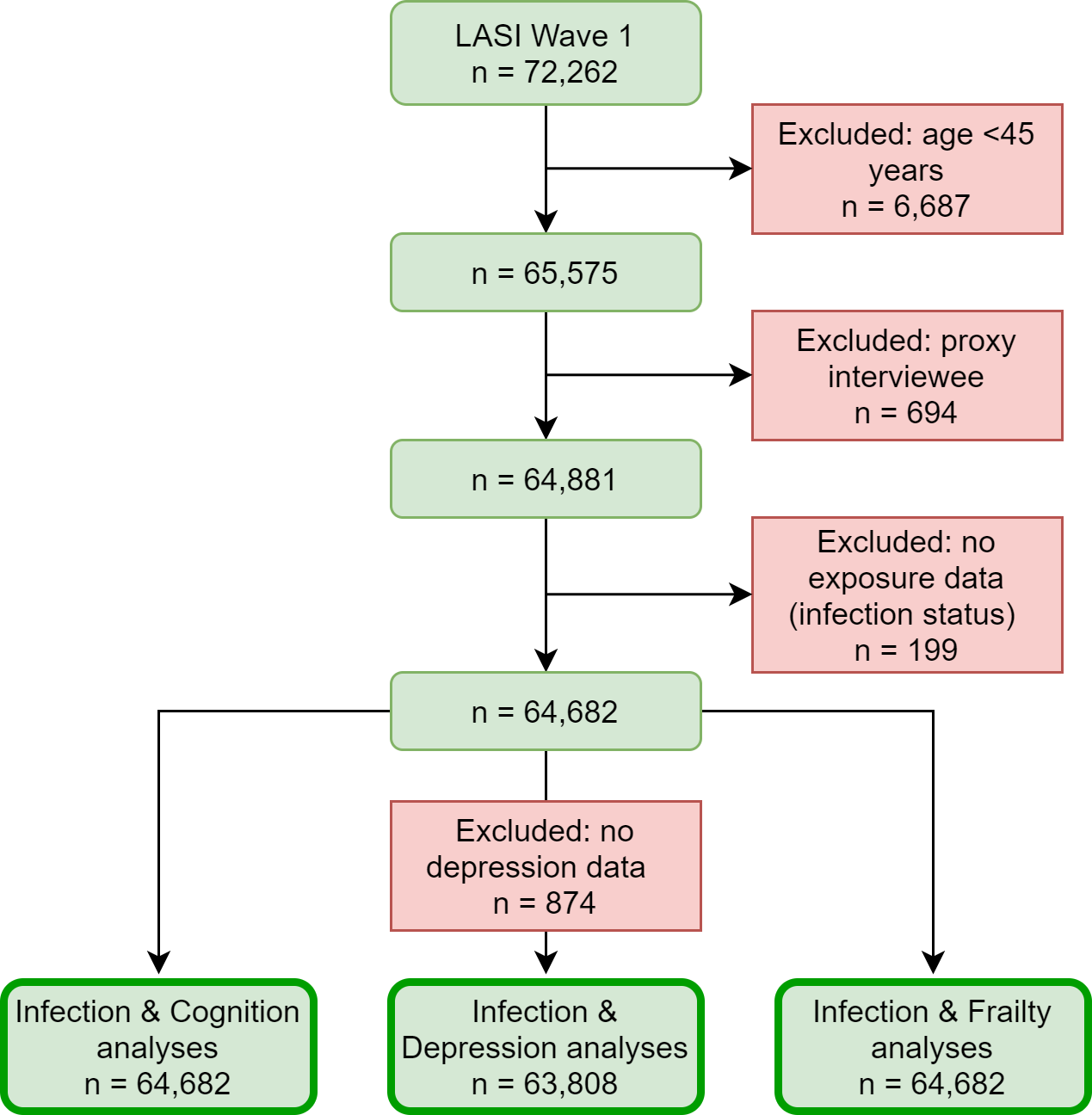


Figure 1: Flow chart for inclusion and exclusion into study population for analyses on infections and cognition, depression, and frailty risk

Table 1: CES-D-10 Depression questionnaire

|  | **During the past week** | | | |
| --- | --- | --- | --- | --- |
|  | **Rarely or never (less than 1 day)** | **Sometimes**  **(1-2 days)** | **Often**  **(3-4 days)** | **Most or all of the time**  **(5-7 days)** |
| **Had trouble concentrating** | 0 | 1 | 2 | 3 |
| **Felt depressed** | 0 | 1 | 2 | 3 |
| **Everything an effort** | 0 | 1 | 2 | 3 |
| **Felt tired or low energy** | 0 | 1 | 2 | 3 |
| **Was happy*** | 3 | 2 | 1 | 0 |
| **Felt lonely** | 0 | 1 | 2 | 3 |
| **Felt overall satisfied*** | 3 | 2 | 1 | 0 |
| **Felt hopeful about the future*** | 3 | 2 | 1 | 0 |
| **Bothered by little things** | 0 | 1 | 2 | 3 |
| **Felt afraid of something** | 0 | 1 | 2 | 3 |
| *coding reversed for the three questions ascertaining positive feelings | | | | |

Table 2: Domains and deficits coded within the frailty index

| **Domain** |  | **Deficit** | **Cut-off** |
| --- | --- | --- | --- |
| **Activities of daily living (ADLs)** | 1 | difficulty dressing | No = 0, Yes = 1 |
|  | 2 | difficulty walking across a room | No = 0, Yes = 1 |
|  | 3 | difficulty bathing or showering | No = 0, Yes = 1 |
|  | 4 | difficulty getting in and out of bed | No = 0, Yes = 1 |
|  | 5 | difficulty using the toilet | No = 0, Yes = 1 |
|  | 6 | difficulty eating | No = 0, Yes = 1 |
| **Co-morbidities** | 7 | high blood pressure/ hypertension | No = 0, Yes = 1 |
|  | 8 | heart problems | No = 0, Yes = 1 |
|  | 9 | Diabetes | No = 0, Yes = 1 |
|  | 10 | stroke history | No = 0, Yes = 1 |
|  | 11 | lung disease | No = 0, Yes = 1 |
|  | 12 | Asthma | No = 0, Yes = 1 |
|  | 13 | arthritis/ osteoporosis | No = 0, Yes = 1 |
|  | 14 | Cancer | No = 0, Yes = 1 |
|  | 15 | psychiatric disease | No = 0, Yes = 1 |
|  | 16 | Cataracts | No = 0, Yes = 1 |
| **Instrumental activities of daily living (IADLs)** | 17 | difficulty making telephone calls | No = 0, Yes = 1 |
|  | 18 | difficulty taking medications | No = 0, Yes = 1 |
|  | 19 | difficulty using the map to figure out to get around | No = 0, Yes = 1 |
|  | 20 | difficulty preparing a hot meal | No = 0, Yes = 1 |
|  | 21 | difficulty shopping for groceries | No = 0, Yes = 1 |
|  | 22 | difficulty managing money | No = 0, Yes = 1 |
|  | 23 | difficulty doing work around the house and garden | No = 0, Yes = 1 |
| **Mobility** | 24 | difficulty walking 100 yards | No = 0, Yes = 1 |
|  | 25 | difficulty sitting for 2 hours | No = 0, Yes = 1 |
|  | 26 | difficulty getting up from a chair after sitting | No = 0, Yes = 1 |
|  | 27 | difficulty climbing one flight of stairs | No = 0, Yes = 1 |
|  | 28 | difficulty stooping, kneeling, or crouching | No = 0, Yes = 1 |
|  | 29 | difficulty reaching or extending arms above shoulders | No = 0, Yes = 1 |
|  | 30 | difficulty pulling or pushing large objects | No = 0, Yes = 1 |
|  | 31 | difficulty picking up 5p coin from table | No = 0, Yes = 1 |
|  | 32 | difficulty lifting/ carrying 10lbs | No = 0, Yes = 1 |
| **Self-reported Health** | 33 | self-reported general health | No = 0, Yes = 1 |
|  | 34 | measured depression | No = 0, Yes = 1 |
|  | 35 | feels unrested during the day | No = 0, Yes = 1 |
|  | 36 | self-reported poor eyesight | No = 0, Yes = 1 |
|  | 37 | ever had hearing/ear-related problem/condition | No = 0, Yes = 1 |

Table 3: Structure of the models developed for cognition, depression, and frailty analyses

| **Model #** | **Covariate group** | **Cognition** | **Depression** | **Frailty** | **Covariate description** |
| --- | --- | --- | --- | --- | --- |
| 1 | **Demographic** | Sex | Sex | Sex | ‘male’ or ‘female’ |
|  |  | Age | Age | Age | Continuous variable (years) |
| 2 | **Social and Environmental** | Poverty | Poverty | Poverty | Above or below the International Poverty Line (household income $1.90 per person per day) |
|  |  | Caste | Caste | Caste | 1) scheduled caste (SC), scheduled tribe (ST), or “other backwards castes” (OBC)  2) other or no caste, with the SC/ST/OBC population largely deprived of privileges, historically and socially marginalized, and vulnerable |
|  |  | Marital  status | Marital  status | Marital  status | 1) married/partnered  2) not married (including widowed, never married, separated, divorced). |
|  |  | Cooking  fuel | Cooking  fuel | Cooking  fuel | ‘clean’ (liquefied petroleum gas (LPG), biogas, or electric)  ‘unclean’ (kerosene, charcoal/lignite/coal, crop residue, wood/shrub, dung cake). |
|  |  | Improved HH sanitation | Improved HH sanitation | Improved HH sanitation | `improved sanitation’ (flush or pour flush toilet connected to a piped sewer system, septic tank, or pit latrine, or somewhere else)  `unimproved sanitation’ (if the housing respondent reports a flush or pour flush toilet that flushes to somewhere besides a piped sewer system, septic tank, or pit latrine; if the household reports the use of other or no facility, use open space or field; or if the household shares their toilet facility with any other households, regardless of the type of toilet facility) |
|  |  | Education | Education | Education | 1) none/less than primary, 2) primary, 3) middle school, 4) secondary, 5) Diploma/graduate/post-graduate/professional qualification. |
|  |  | Weekly social contact | Weekly social contact |  | Whether the participant has at least weekly social contact with relatives or friends (other than those they live with) in person: assigned a code of 0 if the respondent visits relatives or friends several times a month, at least once a month, rarely/once in a year/ or never/not relevant. Assigned a code of 1 if the respondent visits relatives or friends daily, several times a week, or once a week. |
| 3 | **Lifestyle** | Alcohol  Consumption | Alcohol  Consumption | Alcohol  Consumption | Whether a participant drinks alcohol, if `yes ‘frequency (infrequently [<3 days/month]/frequently [≥1 days/week]) |
|  |  | Smoking | Smoking | Smoking | Has never/has ever smoked |
|  |  |  | BMI |  | Categorised as per guidelines for South Asians into ‘underweight’ (BMI<18.5), ‘normal’ (BMI≥18.5 & <23), ‘overweight’ (BMI ≥23 & <25), ‘obese’ (BMI ≥25).^1^ |
| 4 | **Chronic health conditions** | Frailty | Frailty |  | Frail (FI ≥ 0.2), not frail (<0.21) |
|  | **Potential confounders** | Anaemia | Anaemia | Anaemia | 1) reported having had anaemia during the past two years  2) did not report anaemia during the past two years |
|  | **Potential effect modifiers** | Interaction with HC system | Interaction with HC system | Interaction with HC system | Whether or not respondents reported having received health care or consulted a health care provide (including doctor, nurse/midwife, physiotherapist, pharmacist, traditional/folk healer, dentist and other health care providers) in the past year |

Table 4: Unweighted characteristics of the LASI respondents by outcome and infection status (results are N (%) unless otherwise stated)

|  | **Cognition** | | **Depression** | | **Frailty** | |
| --- | --- | --- | --- | --- | --- | --- |
| **Characteristic** | **No infection, N = 43,135^1^** | **At least one infection, N = 21,547^1^** | **No infection, N = 42,550^1^** | **At least one infection, N = 21,258^1^** | **No infection, N = 43,135^1^** | **At least one infection, N = 21,547^1^** |
| **Age (yrs) (median [IQR])** | 58.00 [50.00-67.00] | 59.00 [51.00-66.00] | 58.00 [50.00-67.00] | 59.00 [51.00-66.00] | 58.00 [50.00-67.00] | 59.00 [51.00-66.00] |
| **Sex** |  |  |  |  |  |  |
| Male | 20,505 (47.5) | 9,549 (44.3) | 20,183 (47.4) | 9,387 (44.2) | 20,505 (47.5) | 9,549 (44.3) |
| Female | 22,630 (52.5) | 11,998 (55.7) | 22,367 (52.6) | 11,871 (55.8) | 22,630 (52.5) | 11,998 (55.7) |
| **International Poverty Line^2^** |  |  |  |  |  |  |
| Above int poverty line | 35,981 (83.4) | >17,724 (>82.3) | 35,472 (83.4) | >17,479 (>82.2) | 35,981 (83.4) | >17,724 (>82.3) |
| Below int poverty line | 7,154 (16.6) | 3,773 (17.5) | 7,078 (16.6) | 3,729 (17.5) | 7,154 (16.6) | 3,773 (17.5) |
| (Missing) | 0 | <50 (<0.1) | 0 | <50 (<0.1) | 0 | <50 (<0.1) |
| **Caste^3^** |  |  |  |  |  |  |
| Scheduled caste (SC)/Scheduled tribe (ST)/ Other backwards tribe (OBT) | 30,591 (71.6) | 15,808 (73.7) | 30,187 (71.6) | 15,606 (73.8) | 30,591 (71.6) | 15,808 (73.7) |
| No caste or other | 12,157 (28.4) | 5,640 (26.3) | 11,982 (28.4) | 5,553 (26.2) | 12,157 (28.4) | 5,640 (26.3) |
| (Missing) | 387 | 99 | 381 | 99 | 387 | 99 |
| **Marital status** |  |  |  |  |  |  |
| Married/partnered | >32,399 (>75.1) | 16,342 (75.8) | >31,947 (>75.1) | 16,132 (75.9) | >32,399 (>75.1) | 16,342 (75.8) |
| Not married | 10,686 (24.8) | 5,205 (24.2) | 10,553 (24.8) | 5,126 (24.1) | 10,686 (24.8) | 5,205 (24.2) |
| (Missing) | <50 (<0.1) | 0 | <50 (<0.1) | 0 | <50 (<0.1) | 0 |
| **Weekly contact with friends/family^4^** | 6,110 (14.3) | 2,650 (12.4) | >6,095 (>14.3) | >2,619 (>12.3) | 6,110 (14.3) | 2,650 (12.4) |
| (Missing) | 520 | 244 | <50 (<0.1) | <50 (<0.1) | 520 | 244 |
| **Education** |  |  |  |  |  |  |
| None/less than primary | >23,728 (>55.0) | 13,898 (64.5) | >23,429 (>55.1) | 13,727 (64.6) | >23,728 (>55.0) | 13,898 (64.5) |
| Primary | 5,865 (13.6) | 2,677 (12.4) | 5,775 (13.6) | 2,636 (12.4) | 5,865 (13.6) | 2,677 (12.4) |
| Middle school | 4,301 (10.0) | 1,888 (8.8) | 4,249 (10.0) | 1,859 (8.7) | 4,301 (10.0) | 1,888 (8.8) |
| Secondary | 6,331 (14.7) | 2,263 (10.5) | 6,237 (14.7) | 2,229 (10.5) | 6,331 (14.7) | 2,263 (10.5) |
| Diploma/grad/post-grad/prof qualification | 2,860 (6.6) | 821 (3.8) | 2,810 (6.6) | 807 (3.8) | 2,860 (6.6) | 821 (3.8) |
| (Missing) | <50 (<0.1) | 0 | <50 (<0.1) | 0 | <50 (<0.1) | 0 |
| **Household uses clean cooking fuel^5^** | 24,395 (57.7) | 9,732 (46.0) | 24,058 (57.6) | 9,594 (45.9) | 24,395 (57.7) | 9,732 (46.0) |
| (Missing) | 851 | 392 | 789 | 369 | 851 | 392 |
| **Household has Improved Sanitation^6^** | 31,732 (75.1) | 14,068 (66.5) | 31,326 (75.0) | 13,902 (66.6) | 31,732 (75.1) | 14,068 (66.5) |
| (Missing) | 857 | 403 | 797 | 380 | 857 | 403 |
| **Drinks Alcohol^7^** |  |  |  |  |  |  |
| Never | 38,424 (89.7) | 19,230 (89.6) | >38,098 (>89.5) | >19,008 (>89.4) | 38,424 (89.7) | 19,230 (89.6) |
| Infrequently | 2,472 (5.8) | 1,417 (6.6) | 2,457 (5.8) | 1,400 (6.6) | 2,472 (5.8) | 1,417 (6.6) |
| Frequently | 1,959 (4.6) | 806 (3.8) | 1,945 (4.6) | 800 (3.8) | 1,959 (4.6) | 806 (3.8) |
| (Missing) | 280 | 94 | <50 (<0.1) | <50 (<0.1) | 280 | 94 |
| **Ever smoked** | 7,336 (17.1) | 4,432 (20.7) | >7,273 (>17.1) | >4,345 (>20.4) | 7,336 (17.1) | 4,432 (20.7) |
| (Missing) | 285 | 104 | <50 (<0.1) | <50 (<0.1) | 285 | 104 |
| **BMI Category** |  |  |  |  |  |  |
| Underweight (BMI <18.5) | 6,440 (16.5) | 4,525 (22.6) | 6,405 (16.5) | 4,489 (22.6) | 6,440 (16.5) | 4,525 (22.6) |
| Normal (BMI >=18.5 & <23) | 14,272 (36.5) | 7,679 (38.4) | 14,182 (36.5) | 7,623 (38.4) | 14,272 (36.5) | 7,679 (38.4) |
| Overweight (BMI >=23 & <25) | 6,166 (15.8) | 2,746 (13.7) | 6,132 (15.8) | 2,728 (13.7) | 6,166 (15.8) | 2,746 (13.7) |
| Obese (BMI >=25) | 12,192 (31.2) | 5,043 (25.2) | 12,122 (31.2) | 5,007 (25.2) | 12,192 (31.2) | 5,043 (25.2) |
| (Missing) | 4,065 | 1,554 | 3,709 | 1,411 | 4,065 | 1,554 |
| **Anaemia^8^** | >753 (>1.7) | >1,714 (>8.0) | >725 (>1.7) | >1,691 (>8.0) | >753 (>1.7) | >1,714 (>8.0) |
| (Missing) | <50 (<0.1) | <50 (<0.1) | <50 (<0.1) | <50 (<0.1) | <50 (<0.1) | <50 (<0.1) |
| **Medical visit^9^** | 21,158 (49.5) | 13,082 (61.2) | 21,005 (49.5) | >12,961 (>61.0) | 21,158 (49.5) | 13,082 (61.2) |
| (Missing) | 385 | 158 | 98 | <50 (<0.1) | 385 | 158 |
| ^1^Median [25%-75%]; n (%) | | | | | | |
| ^2^Whether the household falls above or at/below the international poverty line as defined by the World Bank which is currently set at $1.90 per person per day in 2011 purchasing power parity (PPP) dollars | | | | | | |
| ^3^SC/ST/OBC population is largely deprived of privileges, historically and socially marginalized, and vulnerable | | | | | | |
| ^4^Atleast weekly contact with any of their friends or relatives in person | | | | | | |
| ^5^Clean cooking fuel includes housing respondent reporting their main source of cooking fuel is liquefied petroleum gas (LPG), biogas, or electric | | | | | | |
| ^6^Improved sanitation includes flush or pour flush toilet that flushes to a piped sewer system, flushes to a septic tank, or flushes to pit latrine, or if the housing respondent reports a twin pit/composting toilet or pit latrine, and the housing respondent reports that the household does not share their toilet facility with any other households | | | | | | |
| ^7^Infrequently drinks alcohol: drinks less than three days per month. Frequently drinks alcohol: drinks one or more days per week | | | | | | |
| ^8^Whether the respondent has had anaemia in the past two years | | | | | | |
| ^9^Whether the respondent received health care from or consulted with a health care provider (including home visits) in the past year (i.e. outpatient visits) | | | | | | |
| NB: to prevent disclosure we have used primary suppression to directly suppress cells with small counts (i.e. <50), and secondary suppression to suppress additional cells that do not have small counts themselves but which need to be suppressed to protect the values in the primarily suppressed cells. | | | | | | |

Table 5: Unweighted characteristics of the LASI respondents included in and excluded from the cognition analyses due to missing covariate data, by infection status

|  | **No infection** | | **At least one infection** | |
| --- | --- | --- | --- | --- |
| **Characteristic** | **Excluded, N = 1,749^1^** | **Included, N = 41,386^1^** | **Excluded, N = 740^1^** | **Included, N = 20,807^1^** |
| **Age (yrs)** | 59.00 [51.00-67.00] | 58.00 [50.00-67.00] | 59.00 [50.00-67.00] | 59.00 [51.00-66.00] |
| **Sex** |  |  |  |  |
| Male | 860 (49.2) | 19,645 (47.5) | 336 (45.4) | 9,213 (44.3) |
| Female | 889 (50.8) | 21,741 (52.5) | 404 (54.6) | 11,594 (55.7) |
| **International Poverty Line^2^** |  |  |  |  |
| Above int poverty line | 1,532 (87.6) | 34,449 (83.2) | >585 (>79.1) | 17,139 (82.4) |
| Below int poverty line | 217 (12.4) | 6,937 (16.8) | 105 (14.2) | 3,668 (17.6) |
| (Missing) |  |  | <50 (<0.1) | 0 |
| **Caste^3^** |  |  |  |  |
| Scheduled caste (SC)/Scheduled tribe (ST)/Other backwards tribe (OBT) | 931 (68.4) | 29,660 (71.7) | 442 (69.0) | 15,366 (73.9) |
| No caste or other | 431 (31.6) | 11,726 (28.3) | 199 (31.0) | 5,441 (26.1) |
| (Missing) | 387 | 0 | 99 | 0 |
| **Marital status** |  |  |  |  |
| Married/partnered | >1,250 (>71.5) | 31,149 (75.3) | 553 (74.7) | 15,789 (75.9) |
| Not married | 449 (25.7) | 10,237 (24.7) | 187 (25.3) | 5,018 (24.1) |
| (Missing) | <50 (<0.1) | 0 |  |  |
| ^1^Median [25%-75%]; n (%) | | | | |
| ^2^Whether the household falls above or at/below the international poverty line as defined by the World Bank which is currently set at $1.90 per person per day in 2011 purchasing power parity (PPP) dollars | | | | |
| ^3^SC/ST/OBC population is largely deprived of privileges, historically and socially marginalized, and vulnerable | | | | |

Table 6: Unweighted characteristics of the LASI respondents included in and excluded from the depression analyses due to missing covariate data, by infection status

|  | **No infection** | | **At least one infection** | |
| --- | --- | --- | --- | --- |
| **Characteristic** | **Excluded, N = 5,361^1^** | **Included, N = 37,774^1^** | **Excluded, N = 2,153^1^** | **Included, N = 19,394^1^** |
| **Age (yrs)** | 59.00 [51.00-68.00] | 58.00 [50.00-67.00] | 60.00 [51.00-68.00] | 58.00 [51.00-66.00] |
| **Sex** |  |  |  |  |
| Male | 2,615 (48.8) | 17,890 (47.4) | 998 (46.4) | 8,551 (44.1) |
| Female | 2,746 (51.2) | 19,884 (52.6) | 1,155 (53.6) | 10,843 (55.9) |
| **International Poverty Line^2^** |  |  |  |  |
| Above int poverty line | 4,573 (85.3) | 31,408 (83.1) | >1,771 (>82.3) | 15,953 (82.3) |
| Below int poverty line | 788 (14.7) | 6,366 (16.9) | 332 (15.4) | 3,441 (17.7) |
| (Missing) |  |  | <50 (<0.1) | 0 |
| **Caste^3^** |  |  |  |  |
| Scheduled caste (SC)/Scheduled tribe (ST)/Other backwards tribe (OBT) | 3,365 (67.7) | 27,226 (72.1) | 1,440 (70.1) | 14,368 (74.1) |
| No caste or other | 1,609 (32.3) | 10,548 (27.9) | 614 (29.9) | 5,026 (25.9) |
| (Missing) | 387 | 0 | 99 | 0 |
| **Marital status** |  |  |  |  |
| Married/partnered | >3,909 (>72.9) | 28,490 (75.4) | 1,588 (73.8) | 14,754 (76.1) |
| Not married | 1,402 (26.2) | 9,284 (24.6) | 565 (26.2) | 4,640 (23.9) |
| (Missing) | <50 (<0.1) | 0 |  |  |
| ^1^Median [25%-75%]; n (%) | | | | |
| ^2^Whether the household falls above or at/below the international poverty line as defined by the World Bank which is currently set at $1.90 per person per day in 2011 purchasing power parity (PPP) dollars | | | | |
| ^3^SC/ST/OBC population is largely deprived of privileges, historically and socially marginalized, and vulnerable | | | | |

Table 7: Unweighted characteristics of the LASI respondents included in and excluded from the frailty analyses due to missing covariate data, by infection status

|  | **No infection** | | **At least one infection** | |
| --- | --- | --- | --- | --- |
| **Characteristic** | **Excluded, N = 1,497^1^** | **Included, N = 41,638^1^** | **Excluded, N = 592^1^** | **Included, N = 20,955^1^** |
| **Age (yrs)** | 59.00 [51.00-67.00] | 58.00 [50.00-67.00] | 59.00 [50.00-68.00] | 59.00 [51.00-66.00] |
| **Sex** |  |  |  |  |
| Male | 718 (48.0) | 19,787 (47.5) | 255 (43.1) | 9,294 (44.4) |
| Female | 779 (52.0) | 21,851 (52.5) | 337 (56.9) | 11,661 (55.6) |
| **International Poverty Line^2^** |  |  |  |  |
| Above int poverty line | 1,317 (88.0) | 34,664 (83.3) | >456 (>77.0) | 17,268 (82.4) |
| Below int poverty line | 180 (12.0) | 6,974 (16.7) | 86 (14.6) | 3,687 (17.6) |
| (Missing) |  |  | <50 (<0.1) | 0 |
| **Caste^3^** |  |  |  |  |
| Scheduled caste (SC)/Scheduled tribe (ST)/Other backwards tribe (OBT) | 760 (68.5) | 29,831 (71.6) | 325 (65.9) | 15,483 (73.9) |
| No caste or other | 350 (31.5) | 11,807 (28.4) | 168 (34.1) | 5,472 (26.1) |
| (Missing) | 387 | 0 | 99 | 0 |
| **Marital status** |  |  |  |  |
| Married/partnered | >1,063 (>71.0) | 31,336 (75.3) | 441 (74.5) | 15,901 (75.9) |
| Not married | 384 (25.7) | 10,302 (24.7) | 151 (25.5) | 5,054 (24.1) |
| (Missing) | <50 (<0.1) | 0 |  |  |
| ^1^Median [25%-75%]; n (%) | | | | |
| ^2^Whether the household falls above or at/below the international poverty line as defined by the World Bank which is currently set at $1.90 per person per day in 2011 purchasing power parity (PPP) dollars | | | | |
| ^3^SC/ST/OBC population is largely deprived of privileges, historically and socially marginalized, and vulnerable | | | | |

Table 8: Total number of infections reported by participants

| **Number of infections** | **Prevalence % (95% CI)** |
| --- | --- |
| 0 | 64.7% (95% CI:64.2, 65.2) |
| 1 | 23.4% (95% CI:23.0, 23.9) |
| 2 | 8.2% (95% CI:7.9, 8.5) |
| 3 | 2.5% (95% CI:2.4, 2.7) |
| 4 | 0.8% (95% CI:0.7, 0.9) |
| 5 | 0.2% (95% CI:0.2, 0.3) |
| 6 | 0.04% (95% CI:0.03, 0.07) |
| 7 | 0.03% (95% CI:0.02, 0.06) |
| 8 | 0.10% (95% CI:0.07, 0.14) |
| 9 | 0.01% (95% CI:0.00, 0.02) |

Table 9: Association between infections and impaired cognition in people with at least one infection and those without an infection

|  | **Model 1**   n=64,682 | | **Model 2**   n=62,251 | | **Model 3**   n=62,193 | | **Model 4**   n=62,193 | |
| --- | --- | --- | --- | --- | --- | --- | --- | --- |
| **Characteristic** | **OR**^1^ | **95% CI**^1^ | **OR**^1^ | **95% CI**^1^ | **OR**^1^ | **95% CI**^1^ | **OR**^1^ | **95% CI**^1^ |
| **Any infection** | 1.01 | 0.95, 1.08 | 0.84 | 0.78, 0.90 | 0.84 | 0.79, 0.91 | 0.80 | 0.74, 0.86 |
| **Age (yrs)** | 1.00 | 1.00, 1.00 | 0.99 | 0.98, 0.99 | 0.99 | 0.98, 0.99 | 0.98 | 0.97, 0.98 |
| **Sex** | 3.11 | 2.88, 3.35 | 2.07 | 1.91, 2.25 | 2.30 | 2.09, 2.54 | 2.21 | 2.00, 2.44 |
| **International Poverty Line^2^** |  |  | 1.10 | 1.01, 1.19 | 1.10 | 1.01, 1.19 | 1.11 | 1.02, 1.20 |
| **Caste^3^** |  |  | 0.80 | 0.73, 0.87 | 0.82 | 0.74, 0.89 | 0.80 | 0.73, 0.88 |
| **Marital status** |  |  | 1.34 | 1.24, 1.45 | 1.33 | 1.23, 1.44 | 1.29 | 1.19, 1.40 |
| **Household uses clean cooking fuel^4^** |  |  | 0.61 | 0.56, 0.66 | 0.61 | 0.57, 0.66 | 0.61 | 0.57, 0.66 |
| **Household has Improved Sanitation^5^** |  |  | 0.81 | 0.76, 0.87 | 0.81 | 0.75, 0.87 | 0.81 | 0.75, 0.87 |
| **Education** |  |  |  |  |  |  |  |  |
| None/less than primary |  |  | — | — | — | — | — | — |
| Primary |  |  | 0.17 | 0.14, 0.20 | 0.17 | 0.14, 0.20 | 0.17 | 0.14, 0.20 |
| Middle school |  |  | 0.08 | 0.06, 0.11 | 0.08 | 0.06, 0.11 | 0.08 | 0.06, 0.11 |
| Secondary |  |  | 0.03 | 0.02, 0.05 | 0.03 | 0.02, 0.05 | 0.03 | 0.02, 0.05 |
| Diploma/grad/post-grad/prof qualification |  |  | 0.01 | 0.00, 0.03 | 0.01 | 0.00, 0.03 | 0.01 | 0.00, 0.03 |
| **Weekly contact with friends/family^6^** |  |  | 0.91 | 0.80, 1.03 | 0.91 | 0.80, 1.03 | 0.93 | 0.82, 1.05 |
| **Drinks Alcohol^7^** |  |  |  |  |  |  |  |  |
| Never |  |  |  |  | — | — | — | — |
| Infrequently |  |  |  |  | 1.48 | 1.26, 1.75 | 1.52 | 1.29, 1.79 |
| Frequently |  |  |  |  | 1.59 | 1.35, 1.89 | 1.65 | 1.40, 1.96 |
| **Ever smoked** |  |  |  |  | 1.02 | 0.91, 1.14 | 1.02 | 0.91, 1.14 |
| **Frail** |  |  |  |  |  |  | 1.77 | 1.63, 1.92 |
| ^1^OR = Odds Ratio, CI = Confidence Interval | | | | | | | | |
| ^2^Whether the household falls above or at/below the international poverty line as defined by the World Bank which is currently set at $1.90 per person per day in 2011 purchasing power parity (PPP) dollars | | | | | | | | |
| ^3^SC/ST/OBC population is largely deprived of privileges, historically and socially marginalized, and vulnerable | | | | | | | | |
| ^4^Clean cooking fuel includes housing respondent reporting their main source of cooking fuel is liquefied petroleum gas (LPG), biogas, or electric | | | | | | | | |
| ^5^Improved sanitation includes flush or pour flush toilet that flushes to a piped sewer system, flushes to a septic tank, or flushes to pit latrine, or if the housing respondent reports a twin pit/composting toilet or pit latrine, and the housing respondent reports that the household does not share their toilet facility with any other households | | | | | | | | |
| ^6^Atleast weekly contact with any of their friends or relatives in person | | | | | | | | |
| ^7^Infrequently drinks alcohol: drinks less than three days per month. Frequently drinks alcohol: drinks one or more days per week | | | | | | | | |

Table 10: Association between infections and Global Cognitive Function Z-Score in people with at least one infection and those without an infection

|  | **Model 1** | | **Model 2** | | **Model 3** | | **Model 4** | |
| --- | --- | --- | --- | --- | --- | --- | --- | --- |
| **Characteristic** | **Beta** | **95% CI^1^** | **Beta** | **95% CI^1^** | **Beta** | **95% CI^1^** | **Beta** | **95% CI^1^** |
| **Any infection** | -0.06 | -0.08, -0.05 | 0.03 | 0.01, 0.04 | 0.02 | 0.01, 0.04 | 0.04 | 0.03, 0.05 |
| **Age (yrs)** | -0.02 | -0.02, -0.02 | -0.01 | -0.02, -0.01 | -0.01 | -0.02, -0.01 | -0.01 | -0.01, -0.01 |
| **Sex** | -0.48 | -0.49, -0.46 | -0.26 | -0.27, -0.25 | -0.28 | -0.30, -0.27 | -0.27 | -0.28, -0.25 |
| **International Poverty Line^2^** |  |  | -0.05 | -0.07, -0.03 | -0.05 | -0.07, -0.03 | -0.05 | -0.07, -0.04 |
| **Caste** |  |  | 0.03 | 0.02, 0.05 | 0.03 | 0.01, 0.04 | 0.03 | 0.02, 0.04 |
| **Marital status** |  |  | -0.11 | -0.12, -0.09 | -0.11 | -0.12, -0.09 | -0.10 | -0.11, -0.08 |
| **Household uses clean cooking fuel^3^** |  |  | 0.17 | 0.16, 0.19 | 0.17 | 0.16, 0.18 | 0.17 | 0.15, 0.18 |
| **Household has Improved Sanitation^4^** |  |  | 0.09 | 0.08, 0.11 | 0.09 | 0.08, 0.11 | 0.09 | 0.08, 0.11 |
| **Education** |  |  |  |  |  |  |  |  |
| None/less than primary |  |  | — | — | — | — | — | — |
| Primary |  |  | 0.50 | 0.49, 0.52 | 0.50 | 0.48, 0.52 | 0.50 | 0.48, 0.52 |
| Middle school |  |  | 0.62 | 0.60, 0.64 | 0.62 | 0.60, 0.64 | 0.61 | 0.59, 0.63 |
| Secondary |  |  | 0.78 | 0.76, 0.79 | 0.77 | 0.75, 0.78 | 0.76 | 0.74, 0.77 |
| Diploma/grad/post-grad/prof qualification |  |  | 0.94 | 0.91, 0.96 | 0.92 | 0.90, 0.95 | 0.91 | 0.88, 0.93 |
| **Weekly contact with friends/family^5^** |  |  | 0.04 | 0.02, 0.06 | 0.04 | 0.02, 0.06 | 0.03 | 0.02, 0.05 |
| **Drinks Alcohol^6^** |  |  |  |  |  |  |  |  |
| Never |  |  |  |  | — | — | — | — |
| Infrequently |  |  |  |  | -0.10 | -0.13, -0.07 | -0.11 | -0.13, -0.08 |
| Frequently |  |  |  |  | -0.15 | -0.18, -0.12 | -0.16 | -0.19, -0.13 |
| **Ever smoked** |  |  |  |  | -0.01 | -0.03, 0.01 | -0.01 | -0.03, 0.01 |
| **Frail** |  |  |  |  |  |  | -0.19 | -0.21, -0.18 |
| ^1^CI = Confidence Interval | | | | | | | | |
| ^2^Whether the household falls above or at/below the international poverty line as defined by the World Bank which is currently set at $1.90 per person per day in 2011 purchasing power parity (PPP) dollars | | | | | | | | |
| ^3^Clean cooking fuel includes housing respondent reporting their main source of cooking fuel is liquefied petroleum gas (LPG), biogas, or electric | | | | | | | | |
| ^4^Improved sanitation includes flush or pour flush toilet that flushes to a piped sewer system, flushes to a septic tank, or flushes to pit latrine, or if the housing respondent reports a twin pit/composting toilet or pit latrine, and the housing respondent reports that the household does not share their toilet facility with any other households | | | | | | | | |
| ^5^Atleast weekly contact with any of their friends or relatives in person | | | | | | | | |
| ^6^Infrequently drinks alcohol: drinks less than three days per month. Frequently drinks alcohol: drinks one or more days per week | | | | | | | | |

Table 11: Association between each infection and risk of cognitive impairment, depression, frailty

| **Outcome** | **Infection** | **MODEL 1** | **MODEL 2** | **MODEL 3** | **MODEL 4** |
| --- | --- | --- | --- | --- | --- |
|  |  | **OR (95% CI)** | **OR (95% CI)** | **OR (95% CI)** | **OR (95% CI)** |
| **Cognition** | **Malaria** | 1.22 (1.09, 1.36) | 0.91 (0.81, 1.02) | 0.91 (0.81, 1.02) | 0.89 (0.79, 0.99) |
|  | **Diarrhoea** | 0.91 (0.83, 1.01) | 0.75 (0.68, 0.83) | 0.76 (0.69, 0.84) | 0.73 (0.66, 0.80) |
|  | **Typhoid** | 0.97 (0.85, 1.12) | 0.89 (0.77, 1.03) | 0.90 (0.78, 1.04) | 0.85 (0.74, 0.98) |
|  | **Periodontal** | 1.02 (0.93, 1.11) | 0.88 (0.80, 0.96) | 0.88 (0.81, 0.97) | 0.83 (0.76, 0.91) |
|  | **Jaundice** | 1.44 (1.20, 1.74) | 1.16 (0.95, 1.41) | 1.16 (0.95, 1.41) | 1.08 (0.88, 1.32) |
|  | **TB** | 1.87 (1.38, 2.55) | 1.56 (1.12, 2.16) | 1.58 (1.13, 2.19) | 1.50 (1.07, 2.10) |
|  | **UTI** | 0.96 (0.76, 1.22) | 1.00 (0.78, 1.28) | 1.02 (0.79, 1.30) | 0.90 (0.70, 1.16) |
|  | **Chikungunya** | 0.82 (0.64, 1.04) | 0.94 (0.73, 1.21) | 0.95 (0.74, 1.22) | 0.92 (0.72, 1.18) |
|  | **Dengue** | 0.92 (0.64, 1.31) | 1.02 (0.70, 1.49) | 1.03 (0.71, 1.50) | 1.00 (0.68, 1.46) |
| **Depression** | **Malaria** | 1.36 (1.26, 1.46) | 1.26 (1.16, 1.36) | 1.24 (1.14, 1.34) | 1.19 (1.10, 1.30) |
|  | **Diarrhoea** | 1.28 (1.20, 1.37) | 1.21 (1.14, 1.30) | 1.19 (1.11, 1.28) | 1.11 (1.03, 1.19) |
|  | **Typhoid** | 1.36 (1.24, 1.49) | 1.33 (1.21, 1.46) | 1.32 (1.20, 1.45) | 1.20 (1.09, 1.32) |
|  | **Periodontal** | 1.50 (1.42, 1.59) | 1.47 (1.38, 1.56) | 1.43 (1.34, 1.52) | 1.30 (1.22, 1.38) |
|  | **Jaundice** | 1.57 (1.38, 1.80) | 1.47 (1.29, 1.69) | 1.44 (1.25, 1.66) | 1.28 (1.11, 1.48) |
|  | **TB** | 1.45 (1.16, 1.83) | 1.37 (1.09, 1.73) | 1.30 (1.03, 1.65) | 1.17 (0.92, 1.50) |
|  | **UTI** | 1.63 (1.41, 1.87) | 1.66 (1.44, 1.92) | 1.63 (1.40, 1.89) | 1.33 (1.14, 1.55) |
|  | **Chikungunya** | 1.34 (1.16, 1.54) | 1.41 (1.22, 1.63) | 1.36 (1.17, 1.58) | 1.31 (1.12, 1.53) |
|  | **Dengue** | 1.14 (0.92, 1.43) | 1.22 (0.98, 1.52) | 1.18 (0.93, 1.49) | 1.13 (0.89, 1.44) |
| **Frailty** | **Malaria** | 1.36 (1.24, 1.49) | 1.28 (1.17, 1.40) | 1.28 (1.17, 1.40) |  |
|  | **Diarrhoea** | 1.57 (1.46, 1.69) | 1.51 (1.41, 1.63) | 1.51 (1.40, 1.62) |  |
|  | **Typhoid** | 1.77 (1.59, 1.96) | 1.72 (1.55, 1.91) | 1.72 (1.55, 1.91) |  |
|  | **Periodontal** | 2.00 (1.87, 2.14) | 1.94 (1.81, 2.07) | 1.92 (1.79, 2.06) |  |
|  | **Jaundice** | 2.12 (1.82, 2.49) | 2.00 (1.71, 2.34) | 2.01 (1.72, 2.36) |  |
|  | **TB** | 2.04 (1.57, 2.66) | 1.85 (1.42, 2.43) | 1.87 (1.43, 2.45) |  |
|  | **UTI** | 2.92 (2.51, 3.41) | 2.94 (2.51, 3.43) | 2.94 (2.51, 3.44) |  |
|  | **Chikungunya** | 1.31 (1.11, 1.55) | 1.34 (1.13, 1.59) | 1.34 (1.13, 1.59) |  |
|  | **Dengue** | 1.34 (1.04, 1.73) | 1.37 (1.06, 1.78) | 1.38 (1.06, 1.79) |  |

Table 12: Association between each infection and change in GCF Z-Score, Depression score, Frailty index

| **Outcome** | **Infection** | **MODEL 1** | **MODEL 2** | **MODEL 3** | **MODEL 4** |
| --- | --- | --- | --- | --- | --- |
|  |  | **Score difference (95% CI)** | **Score difference (95% CI)** | **Score difference (95% CI)** | **Score difference (95% CI)** |
| **Cognition (GCF Z-score)** | **Malaria** | -0.13 (-0.15, -0.10) | 0.02 (-0.01, 0.04) | 0.02 (-0.01, 0.04) | 0.02 (0.00, 0.05) |
|  | **Diarrhoea** | -0.03 (-0.05, -0.01) | 0.06 (0.04, 0.08) | 0.06 (0.04, 0.07) | 0.07 (0.05, 0.09) |
|  | **Typhoid** | -0.02 (-0.05, 0.01) | 0.03 (0.01, 0.06) | 0.03 (0.00, 0.06) | 0.05 (0.02, 0.07) |
|  | **Periodontal** | -0.05 (-0.07, -0.03) | 0.03 (0.01, 0.04) | 0.02 (0.01, 0.04) | 0.04 (0.02, 0.06) |
|  | **Jaundice** | -0.17 (-0.22, -0.13) | -0.05 (-0.09, -0.01) | -0.05 (-0.09, -0.01) | -0.03 (-0.07, 0.01) |
|  | **TB** | -0.24 (-0.31, -0.16) | -0.12 (-0.19, -0.06) | -0.13 (-0.19, -0.06) | -0.11 (-0.17, -0.04) |
|  | **UTI** | 0.00 (-0.04, 0.05) | -0.00 (-0.04, 0.04) | -0.00 (-0.04, 0.04) | 0.03 (-0.01, 0.07) |
|  | **Chikungunya** | 0.09 (0.04, 0.14) | 0.03 (-0.01, 0.08) | 0.03 (-0.01, 0.07) | 0.04 (-0.00, 0.08) |
|  | **Dengue** | 0.04 (-0.03, 0.12) | 0.00 (-0.06, 0.06) | 0.00 (-0.06, 0.06) | 0.01 (-0.05, 0.07) |
| **Depression (CES-D-10 score, range 0-30)** | **Malaria** | 0.90 (0.76, 1.04) | 0.66 (0.52, 0.80) | 0.62 (0.48, 0.77) | 0.54 (0.40, 0.68) |
|  | **Diarrhoea** | 0.65 (0.53, 0.77) | 0.46 (0.34, 0.58) | 0.42 (0.30, 0.54) | 0.28 (0.16, 0.39) |
|  | **Typhoid** | 0.76 (0.59, 0.93) | 0.66 (0.49, 0.84) | 0.63 (0.45, 0.81) | 0.45 (0.27, 0.63) |
|  | **Periodontal** | 1.15 (1.04, 1.26) | 1.04 (0.93, 1.15) | 0.97 (0.86, 1.08) | 0.77 (0.66, 0.89) |
|  | **Jaundice** | 1.03 (0.75, 1.30) | 0.80 (0.53, 1.07) | 0.79 (0.51, 1.07) | 0.56 (0.29, 0.83) |
|  | **TB** | 1.04 (0.56, 1.51) | 0.84 (0.36, 1.32) | 0.73 (0.24, 1.22) | 0.53 (0.06, 1.00) |
|  | **UTI** | 0.98 (0.69, 1.28) | 0.99 (0.70, 1.28) | 0.88 (0.59, 1.18) | 0.48 (0.20, 0.77) |
|  | **Chikungunya** | 0.96 (0.69, 1.22) | 1.11 (0.85, 1.38) | 1.11 (0.84, 1.38) | 1.02 (0.76, 1.28) |
|  | **Dengue** | 0.65 (0.24, 1.05) | 0.77 (0.38, 1.16) | 0.75 (0.35, 1.16) | 0.67 (0.26, 1.07) |
| **Frailty (range 0-0.919)** | **Malaria** | 0.020 (0.015, 0.025) | 0.017 (0.012, 0.022) | 0.017 (0.012, 0.022) |  |
|  | **Diarrhoea** | 0.033 (0.029, 0.037) | 0.031 (0.027, 0.035) | 0.031 (0.027, 0.035) |  |
|  | **Typhoid** | 0.042 (0.036, 0.048) | 0.041 (0.035, 0.047) | 0.041 (0.035, 0.047) |  |
|  | **Periodontal** | 0.050 (0.047, 0.054) | 0.049 (0.045, 0.053) | 0.048 (0.045, 0.052) |  |
|  | **Jaundice** | 0.055 (0.046, 0.065) | 0.051 (0.041, 0.060) | 0.051 (0.042, 0.061) |  |
|  | **TB** | 0.062 (0.045, 0.078) | 0.057 (0.040, 0.074) | 0.058 (0.041, 0.075) |  |
|  | **UTI** | 0.083 (0.073, 0.093) | 0.082 (0.072, 0.092) | 0.082 (0.072, 0.092) |  |
|  | **Chikungunya** | 0.021 (0.013, 0.030) | 0.023 (0.014, 0.031) | 0.023 (0.014, 0.031) |  |
|  | **Dengue** | 0.028 (0.015, 0.041) | 0.028 (0.015, 0.041) | 0.028 (0.015, 0.041) |  |

Table 13: Association between infections and depression in people with at least one infection and those without an infection

|  | **Model 1  n=63,808** | | **Model 2  n=62,079** | | **Model 3  n=57,168** | | **Model 4  n=57,168** | |
| --- | --- | --- | --- | --- | --- | --- | --- | --- |
| **Characteristic** | **OR^1^** | **95% CI^1^** | **OR^1^** | **95% CI^1^** | **OR^1^** | **95% CI^1^** | **OR^1^** | **95% CI^1^** |
| **Any infection** | 1.47 | 1.41, 1.54 | 1.42 | 1.36, 1.49 | 1.39 | 1.33, 1.46 | 1.28 | 1.22, 1.35 |
| **Age (yrs)** | 1.01 | 1.01, 1.02 | 1.01 | 1.01, 1.01 | 1.01 | 1.00, 1.01 | 0.99 | 0.99, 0.99 |
| **Sex** | 1.29 | 1.24, 1.35 | 1.12 | 1.06, 1.17 | 1.15 | 1.09, 1.22 | 1.05 | 0.98, 1.11 |
| **International Poverty Line^2^** |  |  | 1.03 | 0.97, 1.10 | 1.02 | 0.96, 1.09 | 1.04 | 0.97, 1.11 |
| **Caste^3^** |  |  | 0.90 | 0.85, 0.95 | 0.91 | 0.86, 0.97 | 0.89 | 0.84, 0.94 |
| **Marital status** |  |  | 1.43 | 1.35, 1.51 | 1.43 | 1.35, 1.52 | 1.38 | 1.30, 1.46 |
| **Household uses clean cooking fuel^4^** |  |  | 0.92 | 0.87, 0.97 | 0.94 | 0.89, 1.00 | 0.97 | 0.91, 1.02 |
| **Household has Improved Sanitation^5^** |  |  | 0.82 | 0.78, 0.87 | 0.84 | 0.79, 0.88 | 0.83 | 0.79, 0.88 |
| **Education** |  |  |  |  |  |  |  |  |
| None/less than primary |  |  | — | — | — | — | — | — |
| Primary |  |  | 0.90 | 0.84, 0.97 | 0.91 | 0.84, 0.98 | 0.92 | 0.85, 1.00 |
| Middle school |  |  | 0.88 | 0.81, 0.97 | 0.89 | 0.81, 0.97 | 0.92 | 0.83, 1.01 |
| Secondary |  |  | 0.76 | 0.70, 0.83 | 0.76 | 0.70, 0.83 | 0.82 | 0.75, 0.89 |
| Diploma/grad/post-grad/prof qualification |  |  | 0.71 | 0.63, 0.81 | 0.75 | 0.66, 0.86 | 0.84 | 0.73, 0.95 |
| **Weekly contact with friends/family^6^** |  |  | 0.96 | 0.89, 1.03 | 0.98 | 0.91, 1.06 | 1.03 | 0.95, 1.11 |
| **Drinks Alcohol^7^** |  |  |  |  |  |  |  |  |
| Never |  |  |  |  | — | — | — | — |
| Infrequently |  |  |  |  | 0.89 | 0.80, 1.00 | 0.92 | 0.82, 1.03 |
| Frequently |  |  |  |  | 0.90 | 0.79, 1.03 | 0.94 | 0.82, 1.08 |
| **Ever smoked** |  |  |  |  | 1.08 | 1.01, 1.16 | 1.08 | 1.00, 1.16 |
| **BMI Category** |  |  |  |  |  |  |  |  |
| Underweight (BMI <18.5) |  |  |  |  | — | — | — | — |
| Normal (BMI >=18.5 & <23) |  |  |  |  | 0.86 | 0.80, 0.92 | 0.86 | 0.81, 0.92 |
| Overweight (BMI >=23 & <25) |  |  |  |  | 0.84 | 0.77, 0.91 | 0.82 | 0.75, 0.89 |
| Obese (BMI >=25) |  |  |  |  | 0.87 | 0.80, 0.93 | 0.81 | 0.75, 0.87 |
| **Frail** |  |  |  |  |  |  | 3.04 | 2.86, 3.23 |
| ^1^OR = Odds Ratio, CI = Confidence Interval | | | | | | | | |
| ^2^Whether the household falls above or at/below the international poverty line as defined by the World Bank which is currently set at $1.90 per person per day in 2011 purchasing power parity (PPP) dollars | | | | | | | | |
| ^3^SC/ST/OBC population is largely deprived of privileges, historically and socially marginalized, and vulnerable | | | | | | | | |
| ^4^Clean cooking fuel includes housing respondent reporting their main source of cooking fuel is liquefied petroleum gas (LPG), biogas, or electric | | | | | | | | |
| ^5^Improved sanitation includes flush or pour flush toilet that flushes to a piped sewer system, flushes to a septic tank, or flushes to pit latrine, or if the housing respondent reports a twin pit/composting toilet or pit latrine, and the housing respondent reports that the household does not share their toilet facility with any other households | | | | | | | | |
| ^6^Atleast weekly contact with any of their friends or relatives in person | | | | | | | | |
| ^7^Infrequently drinks alcohol: drinks less than three days per month. Frequently drinks alcohol: drinks one or more days per week | | | | | | | | |

Table 14: Association between infections and Depression Score in people with at least one infection and those without an infection

|  | **Model 1** | | **Model 2** | | **Model 3** | | **Model 4** | |
| --- | --- | --- | --- | --- | --- | --- | --- | --- |
| **Characteristic** | **Beta** | **95% CI^1^** | **Beta** | **95% CI^1^** | **Beta** | **95% CI^1^** | **Beta** | **95% CI^1^** |
| **Any infection** | 1.0 | 0.96, 1.1 | 0.91 | 0.83, 1.0 | 0.86 | 0.78, 0.95 | 0.70 | 0.62, 0.79 |
| **Age (yrs)** | 0.03 | 0.03, 0.03 | 0.01 | 0.01, 0.02 | 0.01 | 0.01, 0.01 | -0.02 | -0.02, -0.01 |
| **Sex** | 0.57 | 0.49, 0.65 | 0.18 | 0.09, 0.26 | 0.26 | 0.16, 0.36 | 0.09 | -0.01, 0.19 |
| **International Poverty Line^2^** |  |  | 0.10 | 0.00, 0.21 | 0.10 | -0.01, 0.21 | 0.12 | 0.01, 0.23 |
| **Caste** |  |  | -0.23 | -0.32, -0.13 | -0.19 | -0.29, -0.09 | -0.23 | -0.32, -0.13 |
| **Marital status** |  |  | 0.87 | 0.76, 0.97 | 0.87 | 0.76, 0.98 | 0.76 | 0.65, 0.86 |
| **Household uses clean cooking fuel^3^** |  |  | -0.38 | -0.47, -0.29 | -0.30 | -0.39, -0.20 | -0.25 | -0.35, -0.16 |
| **Household has Improved Sanitation^4^** |  |  | -0.39 | -0.48, -0.30 | -0.37 | -0.47, -0.28 | -0.37 | -0.46, -0.27 |
| **Education** |  |  |  |  |  |  |  |  |
| None/less than primary |  |  | — | — | — | — | — | — |
| Primary |  |  | -0.34 | -0.46, -0.21 | -0.33 | -0.46, -0.20 | -0.29 | -0.42, -0.16 |
| Middle school |  |  | -0.25 | -0.40, -0.10 | -0.25 | -0.40, -0.09 | -0.18 | -0.33, -0.03 |
| Secondary |  |  | -0.76 | -0.90, -0.63 | -0.74 | -0.88, -0.60 | -0.61 | -0.75, -0.47 |
| Diploma/grad/post-grad/prof qualification |  |  | -1.0 | -1.2, -0.85 | -0.99 | -1.2, -0.79 | -0.82 | -1.0, -0.62 |
| **Weekly contact with friends/family^5^** |  |  | -0.69 | -0.82, -0.56 | -0.66 | -0.80, -0.53 | -0.59 | -0.72, -0.45 |
| **Drinks Alcohol^6^** |  |  |  |  |  |  |  |  |
| Never |  |  |  |  | — | — | — | — |
| Infrequently |  |  |  |  | -0.12 | -0.29, 0.06 | -0.06 | -0.24, 0.11 |
| Frequently |  |  |  |  | -0.33 | -0.54, -0.11 | -0.25 | -0.47, -0.04 |
| **Ever smoked** |  |  |  |  | 0.19 | 0.08, 0.31 | 0.19 | 0.07, 0.30 |
| **BMI Category** |  |  |  |  |  |  |  |  |
| Underweight (BMI <18.5) |  |  |  |  | — | — | — | — |
| Normal (BMI >=18.5 & <23) |  |  |  |  | -0.29 | -0.41, -0.17 | -0.27 | -0.38, -0.15 |
| Overweight (BMI >=23 & <25) |  |  |  |  | -0.32 | -0.47, -0.18 | -0.34 | -0.48, -0.19 |
| Obese (BMI >=25) |  |  |  |  | -0.36 | -0.50, -0.23 | -0.47 | -0.60, -0.33 |
| **Frail** |  |  |  |  |  |  | 2.1 | 2.0, 2.2 |
| ^1^CI = Confidence Interval | | | | | | | | |
| ^2^Whether the household falls above or at/below the international poverty line as defined by the World Bank which is currently set at $1.90 per person per day in 2011 purchasing power parity (PPP) dollars | | | | | | | | |
| ^3^Clean cooking fuel includes housing respondent reporting their main source of cooking fuel is liquefied petroleum gas (LPG), biogas, or electric | | | | | | | | |
| ^4^Improved sanitation includes flush or pour flush toilet that flushes to a piped sewer system, flushes to a septic tank, or flushes to pit latrine, or if the housing respondent reports a twin pit/composting toilet or pit latrine, and the housing respondent reports that the household does not share their toilet facility with any other households | | | | | | | | |
| ^5^Atleast weekly contact with any of their friends or relatives in person | | | | | | | | |
| ^6^Infrequently drinks alcohol: drinks less than three days per month. Frequently drinks alcohol: drinks one or more days per week | | | | | | | | |

Table 15: Association between infections and frailty in people with at least one infection and those without an infection

|  | **Model 1  n=64,682** | | **Model 2  n=62,934** | | **Model 3  n=62,593** | |
| --- | --- | --- | --- | --- | --- | --- |
| **Characteristic** | **OR^1^** | **95% CI^1^** | **OR^1^** | **95% CI^1^** | **OR^1^** | **95% CI^1^** |
| **Any infection** | 1.81 | 1.71, 1.91 | 1.75 | 1.66, 1.85 | 1.74 | 1.65, 1.84 |
| **Age (yrs)** | 1.09 | 1.09, 1.09 | 1.08 | 1.08, 1.09 | 1.08 | 1.08, 1.09 |
| **Sex** | 2.36 | 2.23, 2.49 | 2.02 | 1.91, 2.15 | 1.94 | 1.81, 2.08 |
| **International Poverty Line^2^** |  |  | 0.93 | 0.87, 1.00 | 0.93 | 0.87, 1.00 |
| **Caste^3^** |  |  | 1.19 | 1.12, 1.26 | 1.18 | 1.11, 1.25 |
| **Marital status** |  |  | 1.22 | 1.14, 1.29 | 1.22 | 1.15, 1.30 |
| **Household uses clean cooking fuel^4^** |  |  | 0.91 | 0.86, 0.97 | 0.91 | 0.86, 0.97 |
| **Household has Improved Sanitation^5^** |  |  | 0.99 | 0.93, 1.05 | 0.99 | 0.93, 1.05 |
| **Education** |  |  |  |  |  |  |
| None/less than primary |  |  | — | — | — | — |
| Primary |  |  | 0.94 | 0.86, 1.02 | 0.93 | 0.85, 1.02 |
| Middle school |  |  | 0.82 | 0.73, 0.91 | 0.80 | 0.72, 0.90 |
| Secondary |  |  | 0.60 | 0.54, 0.67 | 0.60 | 0.54, 0.66 |
| Diploma/grad/post-grad/prof qualification |  |  | 0.46 | 0.40, 0.54 | 0.45 | 0.39, 0.52 |
| **Drinks Alcohol^6^** |  |  |  |  |  |  |
| Never |  |  |  |  | — | — |
| Infrequently |  |  |  |  | 0.74 | 0.64, 0.85 |
| Frequently |  |  |  |  | 0.61 | 0.51, 0.72 |
| **Ever smoked** |  |  |  |  | 1.04 | 0.96, 1.13 |
| ^1^OR = Odds Ratio, CI = Confidence Interval | | | | | | |
| ^2^Whether the household falls above or at/below the international poverty line as defined by the World Bank which is currently set at $1.90 per person per day in 2011 purchasing power parity (PPP) dollars | | | | | | |
| ^3^SC/ST/OBC population is largely deprived of privileges, historically and socially marginalized, and vulnerable | | | | | | |
| ^4^Clean cooking fuel includes housing respondent reporting their main source of cooking fuel is liquefied petroleum gas (LPG), biogas, or electric | | | | | | |
| ^5^Improved sanitation includes flush or pour flush toilet that flushes to a piped sewer system, flushes to a septic tank, or flushes to pit latrine, or if the housing respondent reports a twin pit/composting toilet or pit latrine, and the housing respondent reports that the household does not share their toilet facility with any other households | | | | | | |
| ^6^Infrequently drinks alcohol: drinks less than three days per month. Frequently drinks alcohol: drinks one or more days per week | | | | | | |

Table 16: Association between infections and Frailty Index in people with at least one infection and those without an infection

|  | **Model 1** | | **Model 2** | | **Model 3** | |
| --- | --- | --- | --- | --- | --- | --- |
| **Characteristic** | **Beta** | **95% CI1** | **Beta** | **95% CI1** | **Beta** | **95% CI1** |
| **Any infection** | 0.041 | 0.038, 0.044 | 0.039 | 0.036, 0.042 | 0.039 | 0.036, 0.042 |
| **Age (yrs)** | 0.006 | 0.006, 0.006 | 0.006 | 0.006, 0.006 | 0.006 | 0.006, 0.006 |
| **Sex** | 0.054 | 0.052, 0.057 | 0.044 | 0.041, 0.047 | 0.043 | 0.040, 0.046 |
| **International Poverty Line^2^** |  |  | -0.007 | -0.011, -0.003 | -0.007 | -0.011, -0.003 |
| **Caste** |  |  | 0.011 | 0.008, 0.014 | 0.010 | 0.007, 0.013 |
| **Marital status** |  |  | 0.020 | 0.017, 0.024 | 0.020 | 0.017, 0.024 |
| **Household uses clean cooking fue^l3^** |  |  | -0.003 | -0.006, -0.001 | -0.003 | -0.006, 0.000 |
| **Household has Improved Sanitation^4^** |  |  | 0.000 | -0.003, 0.003 | 0.000 | -0.003, 0.003 |
| **Education** |  |  |  |  |  |  |
| None/less than primary |  |  | — | — | — | — |
| Primary |  |  | -0.006 | -0.010, -0.002 | -0.006 | -0.010, -0.002 |
| Middle school |  |  | -0.011 | -0.015, -0.007 | -0.012 | -0.016, -0.007 |
| Secondary |  |  | -0.024 | -0.028, -0.021 | -0.025 | -0.028, -0.021 |
| Diploma/grad/post-grad/prof qualification |  |  | -0.036 | -0.041, -0.032 | -0.037 | -0.041, -0.032 |
| **Drinks Alcohol^5^** |  |  |  |  |  |  |
| Never |  |  |  |  | — | — |
| Infrequently |  |  |  |  | -0.011 | -0.016, -0.006 |
| Frequently |  |  |  |  | -0.017 | -0.022, -0.012 |
| **Ever smoked** |  |  |  |  | 0.005 | 0.001, 0.008 |
| **1CI = Confidence Interval** | | | | | | |
| **2Whether the household falls above or at/below the international poverty line as defined by the World Bank which is currently set at $1.90 per person per day in 2011 purchasing power parity (PPP) dollars** | | | | | | |
| **3Clean cooking fuel includes housing respondent reporting their main source of cooking fuel is liquefied petroleum gas (LPG), biogas, or electric** | | | | | | |
| **4Improved sanitation includes flush or pour flush toilet that flushes to a piped sewer system, flushes to a septic tank, or flushes to pit latrine, or if the housing respondent reports a twin pit/composting toilet or pit latrine, and the housing respondent reports that the household does not share their toilet facility with any other households** | | | | | | |
| **5Infrequently drinks alcohol: drinks less than three days per month. Frequently drinks alcohol: drinks one or more days per week** | | | | | | |

Table 17: Results of sensitivity analyses

| **Analysis #** | **Analysis description** | **Analysis justification** | **Main analysis,**  **OR (95% CI)** | **Sensitivity analysis,**  **OR (95% CI)** |
| --- | --- | --- | --- | --- |
| 1.a | Investigating depression as a confounder in the association between any infection and impaired **cognition** | In our main analysis we did not control for depression in the association between infection and impaired cognition/frailty, however, it is possible that depression is associated with both infection and impaired cognition/frailty, therefore we did this extra analysis to see if our results were robust after accounting for depression | 0.80 (0.74-0.86) | 0.80 (0.74-0.86) |
| 1.b | Investigating depression as a confounder in the association between any infection and **frailty** |  | 1.74 (1.65-1.84) | 1.64 (1.55-1.74) |
| 2 | Investigating impaired cognition as a confounder in the association between any infection and **frailty** | In our main analysis we did not control for impaired cognition in the association between infection and frailty, however, it is possible that impaired cognition is associated with both infection and frailty, therefore we did this extra analysis to see if our results were robust after accounting for impaired cognition | 1.74 (1.65-1.84) | 1.76 (1.67-1.86) |
| 3.a | Anaemia as a confounder in the association between **malaria** and impaired **cognition** | In our main analysis we did not control for anaemia in the association between any infection and impaired cognition/depression/frailty however, when investigating the individual infections as exposures, it is possible that anaemia  is associated with both malaria infection and impaired cognition/depression/frailty , therefore we did this extra analysis to see if our results were robust after accounting for anaemia | 0.89 (0.79, 0.99) | 0.90 (0.80-1.01) |
| 3.b | Anaemia as a confounder in the association between **malaria** and **depression** |  | 1.19 (1.10, 1.30) | 1.19 (1.10-1.29) |
| 3.c | Anaemia as a confounder in the association between **malaria** and **frailty** |  | 1.28 (1.17, 1.40) | 1.17 (1.07-1.29) |

Table 18: Association between number of infections and impaired cognition, depression, and frailty

| **Outcome** | **Number of infections** | **Weighted Number of events/ individuals** | **ORs (95% CI)** |
| --- | --- | --- | --- |
| **Impaired Cognition** | **0 infections** | 4,509 /43,565 | Ref |
|  | **1-3 infections** | 2,487/22,994 | 0.80 (0.75-0.87) |
|  | **4+ infections** | 84/789 | 0.70 (0.50-0.98) |
| **Depression** | **0 infections** | 10,624/42,972 | Ref |
|  | **1-3 infections** | 7,361/22,650 | 1.28 (1.22-1.35) |
|  | **4+ infections** | 295/777 | 1.40 (1.13-1.74) |
| **Frailty** | **0 infections** | 7,372/43,565 | Ref |
|  | **1-3 infections** | 5,740/22,994 | 1.69 (1.60-1.79) |
|  | **4+ infections** | 290/789 | 3.60 (2.85-4.53) |

Table 19: Association between attendance at a medical visit during the past year and impaired cognition, depression, and frailty between those with at least one infection relative to those without an infection

| **Outcome** | **Medical visit (past year)** | **Weighted Number of events/individuals** | **Stratified ORs (95% CI)** | **Log reg with interaction term ORs (95% CI)** | **Interaction p-values** |
| --- | --- | --- | --- | --- | --- |
| **Cognition** | **No** | 3,039/28,626 | 0.76 (0.67, 0.85) | 0.78 (0.70, 0.87) | 0.4378 |
|  | **Yes** | 3,976 /3,8190 | 0.84 (0.76, 0.92) | 0.82 (0.75, 0.91) |  |
| **Depression** | **No** | 7,664/28,402 | 1.42 (1.31, 1.54) | 1.39 (1.28, 1.50) | 0.0278 |
|  | **Yes** | 10,576/37,876 | 1.21 (1.13, 1.30) | 1.24 (1.16, 1.32) |  |
| **Frailty** | **No** | 4,365 /28,626 | 2.00 (1.81, 2.20) | 1.98 (1.81, 2.17) | <0.001 |
|  | **Yes** | 8,972/38,190 | 1.53 (1.43, 1.64) | 1.53 (1.43, 1.64) |  |

Table 20: Association between categorical age and impaired cognition, depression, and frailty between those with at least one infection relative to those without an infection

| **Outcome** | **Categorical age** | **Weighted Number of events/individuals** | **Stratified ORs (95% CI)** | **Log reg with interaction term ORs (95% CI)** | **Interaction p-values** |
| --- | --- | --- | --- | --- | --- |
| **Cognition** | **45-59** | 3,981/38,493 | 0.80 (0.73 0.89) | 0.81 (0.73, 0.89) | 0.986 |
|  | **60-75** | 2,441/23,081 | 0.80 (0.71, 0.90) | 0.80 (0.72, 0.90) |  |
|  | **75+** | 657/5,773 | 0.82 (0.66, 1.02) | 0.79 (0.64, 0.98) |  |
| **Depression** | **45-59** | 9,581/37,950 | 1.29 (1.20, 1.38) | 1.32 (1.25, 1.40) | 0.650 |
|  | **60-75** | 6,683/22,772 | 1.31 (1.21, 1.41) | 1.34 (1.26, 1.43) |  |
|  | **75+** | 2,015/5,675 | 1.21 (1.04, 1.41) | 1.25 (1.11, 1.42) |  |
| **Frailty** | **45-59** | 3,922 /38,493 | 1.87 (1.70, 2.05) | 1.91 (1.74, 2.09) | <0.001 |
|  | **60-75** | 6,360 /23,081 | 1.67 (1.55, 1.80) | 1.66 (1.54, 1.79) |  |
|  | **75+** | 3,120/5,773 | 1.39 (1.22, 1.59) | 1.38 (1.20, 1.58) |  |

Table 21: Association between sex and impaired cognition, depression, and frailty between those with at least one infection relative to those without an infection

| **Outcome** | **Sex** | **Weighted Number of events/individuals** | **Stratified ORs**  **(95% CI)** | **Log reg with interaction term ORs (95% CI)** | **Interaction p-values** |
| --- | --- | --- | --- | --- | --- |
| **Cognition** | **Male** | 2,066/35,922 | 0.74 (0.64, 0.86) | 0.76 (0.66, 0.89) | 0.395 |
|  | **Female** | 5,013/31,426 | 0.83 (0.76, 0.90) | 0.82 (0.76, 0.89) |  |
| **Depression** | **Male** | 8,765/35,328 | 1.28 (1.19, 1.39) | 1.28 (1.19, 1.39) | 0.945 |
|  | **Female** | 9,514/31,070 | 1.28 (1.20, 1.37) | 1.28 (1.20, 1.37) |  |
| **Frailty** | **Male** | 4,834/35,922 | 1.80 (1.64, 1.97) | 1.81 (1.66, 1.98) | 0.243 |
|  | **Female** | 8,568/31,426 | 1.70 (1.59, 1.82) | 1.69 (1.58, 1.81) |  |

References

1. Misra A. Ethnic-Specific Criteria for Classification of Body Mass Index: A Perspective for Asian Indians and American Diabetes Association Position Statement. *Diabetes Technology &amp; Therapeutics* 2015;17(9):667-71. doi: 10.1089/dia.2015.0007
